# Efficient removal of Cs^+^ and Sr^2+^ from water using titanate nanotubes embedded in alginate macromolecules

**DOI:** 10.1038/s41598-026-38030-8

**Published:** 2026-02-20

**Authors:** Esraa Farouk, A. H. Zaki, S. I. Eldek, Nabila Shehata

**Affiliations:** 1 Materials Science & Nanotechnology Department, Faculty of Postgraduate Studies for Advanced Sciences (PSAS), Beni-Suef University (BSU), Beni-Suef, Egypt; 2 Environmental Science & Industrial Development Department, Faculty of Postgraduate Studies for Advanced Sciences (PSAS), Beni-Suef University (BSU), Beni-Suef, Egypt

**Keywords:** Adsorption, Cs^+^, Radioactive, Sr^2+^, TNTs, T/G, Water treatment, Chemistry, Environmental sciences, Materials science

## Abstract

The presence of long-lived radionuclides such as Cs⁺ and Sr²⁺ in aquatic systems poses serious environmental and health risks, necessitating the development of highly efficient and practically applicable sorbents. Although various materials have been explored for radionuclide removal, most continue to suffer from slow kinetics, limited selectivity, and difficult recovery. In this study, titanate nanotubes (TNTs) were synthesized hydrothermally and further incorporated into a biodegradable alginate matrix (T/G composite) via ionic gelation to improve handling and solid–liquid separability. The physicochemical features of TNTs and T/G before and after adsorption were examined using XRD, FTIR, FESEM-EDX, zeta potential and N_2_ adsorption/desorption isotherm analyses to better elucidate structural behavior and adsorption mechanisms. Batch experiments evaluated the effects of pH, dosage, initial ion concentration, and contact time. TNTs exhibited rapid adsorption, reaching equilibrium within 15–30 min, with removal efficiencies of 90% for Cs⁺ and 97% for Sr²⁺ at pH 8 and 0.075 g dose. Isotherm fitting using eleven nonlinear models, supported by comprehensive error analysis revealed that both Cs⁺ and Sr²⁺ adsorption followed both Sips and Langmuir–Freundlich models, reflecting heterogeneous and multilayer adsorption behavior. Kinetic results indicated that chemisorption dominated the process, as suggested by the agreement of experimental data with PFO, PSO, MFSO, and Avrami models. The error analysis revealed that the initial concentration of Cs⁺ and Sr²⁺ play a significant role in the adsorption kinetics. The T/G beads achieved removal efficiencies 45–70% Cs⁺ and 70–90% Sr²⁺. Although embedding TNTs in alginate reduced adsorption capacity due to the lower active TNT ratio, it offers excellent mechanical stability and ease of separation. The adsorption mechanism combines electrostatic attraction, surface complexation, and partial ion exchange between Cs⁺/Sr²⁺ and Na⁺ in titanate layers. Regeneration studies demonstrated that TNTs retained over 90% of their initial performance after five cycles, while T/G maintained above 85%, confirming the materials’ reusability. The rapid kinetics, high capacity, and simple regeneration procedure highlight the potential scalability of TNT-based systems for radionuclide removal. However, practical deployment may still require optimization of TNT loading within the alginate matrix and evaluation under real wastewater conditions.

**Supplementary Information:**

The online version contains supplementary material available at 10.1038/s41598-026-38030-8.

## Introduction

Radioactive wastewaters contain hazardous and radiotoxic nuclides such as trans-plutonium elements, fission products with long half-lives, and corrosion products. Radionuclides have long half-life as 28.79, 30.2 and 2 y for ^90^Sr, ^137^Cs and ^134^Cs, respectively, which present serious environmental and health concerns due to their long half-lives, high solubility, and strong affinity for biological systems. Cesium ions disperse readily in soft tissues, while strontium ions accumulate in bones and may induce leukemia or bone sarcoma^1^. Therefore, the development of efficient, selective, and low-cost adsorbents for the removal of Cs⁺ and Sr²⁺ remains a critical requirement in nuclear-related industries, environmental protection, and emergency wastewater treatment. Nevertheless, despite their environmental hazards, certain radionuclides are still used in various practical applications. Certain fission products, such as ^137^Cs and ^90^Sr are now of interest due to their use in agricultural applications, thermoelectric generators in space applications, and radiotracers in industrial and radiotherapy. As a result, a careful consideration has been directed to the detection and management of Cs^+^ and Sr^2+^ in simulation waste solutions since their chemical characteristics are the same as their isotopes^2^. Several techniques have been used for the separation of Cs^+^ and Sr^2+^ from aqueous wastes such as ion exchange, co-precipitation, solvent extraction and adsorption. Ion exchange resins are known for their selectivity; however, their widespread application is limited due to high costs and difficulties in regeneration. Although numerous ion exchangers and sorbents like ammonium molybdophosphate, inorganic materials especially ferrocyanides of transition metal cations, clay minerals, zeolites, zirconium phosphates have been explored for the removal of radionuclides, many of them face limitations, including poor selectivity in the presence of competing ions, slow adsorption rates, limited or inaccessible adsorption sites and challenges in separation and reuse, particularly when dealing with Nano powders. Ferrocyanides demonstrate strong affinity toward Cs⁺ ions but are unstable under acidic conditions. Clays and zeolites offer structural robustness but are hindered by slow diffusion and low adsorption capacity for hydrated Sr²⁺ ions. Adsorbents/ion exchangers by titanium-based materials, especially titanate nanostructures have been deeply investigated for the management of ^90^Sr and ^137^Cs in wastewater. The advantage of this technique is that these materials can resist high temperatures and strong radiation, the exchanged cations are safely disposed and also being highly selective for Cs^+^ and Sr^[2+ 3,4^. Titanium dioxide and titanate nanostructures, particularly nanotubes, are frequently utilized in the treatment of contaminated water because they are non-toxic, low-cost, easy to prepare, efficient, and commercially accessible. Moreover, titanate nanostructure has an essential contribution in the procedure of binding inorganic cations, involving radionuclides, owning to their strong adsorption characteristics^3^. The most common chemical formula of sodium titanate is Na_2_Ti_n_O_2n+1_ (*n* = 6 or 3). However, the structure with *n* = 3 is more traditional as Na^+^ and (Ti_3_O_7_)_2_ bind together, resulting in a layered structure. Moreover, Na_2_Ti_3_O_7_ matrix is considered to have the highest ion-exchange capacity developed from Na₂Ti₃O₇ layered structure. However, depending on the rate of exchange between protons and sodium, the chemical formulas of Na_2_Ti_3_O_7_⋅nH_2_O, Na_2−x_H_x_Ti_3_O_7_⋅nH_2_O, and H_2_Ti_3_O_7_⋅nH_2_O have been suggested for titanate nanotubes (TNTs)^5^. Nanotube is a class of nanoparticles which allow other particles to flow through. Thus, they are long enough to function as a pipe. Titanate nanotubes have been prepared at different hydrothermal conditions. Titanate nanotubes have emerged as an attractive class of sorbent materials for the removal of Cs⁺ and Sr²⁺ from contaminated water. Their exceptionally high surface area, combined with a well-defined tubular architecture, provides multiple accessible diffusion channels that enhance ion transport and uptake. In addition, TNTs possess remarkable mechanical and chemical stability, allowing them to maintain structural integrity even under aggressive environmental or chemical conditions associated with radioactive waste streams. These characteristics, together with their inherently fast adsorption response toward both monovalent and divalent hydrated ions, make TNTs particularly suitable for efficient and reliable remediation of Cs⁺ and Sr²⁺ ^6^. Unfortunately, the applications of nanoparticles in water treatment are limited due to the challenges of separation and recovery from the treated water. The challenges associated with Nano adsorbent separation from solution can be overcome by incorporating them into a polymeric matrix, like alginate as a nanocomposite. Bio-based polymers on the other hand, have several advantages including non-toxicity, biocompatibility, biodegradability, availability, and cost-effectiveness. Furthermore, these hydrophilicity polymers create larger water fluxes than typical synthetic polymers. Hence, they were widely used in water purification. Alginate is a natural polysaccharide extracted from brown algae seaweed with intriguing properties such as non-toxicity, biodegradability, and biocompatibility. It contains many hydroxyl (OH) and carboxyl (COOH) groups in their polymeric structure, making it a good choice for radionuclide removal from aqueous solution^7^. It is formed from β-D-mannuronic acid and 1–4 linked α -L-guluronic blocks. Alginate easily form cross-linked gel matrices when divalent cations are present, particularly Ca^2+^ ions. Hence, these Ca-crosslinked G matrices can be used to prepare gel adsorbents that are more applicable than powder adsorbents. Alginate based composites are considered as effective adsorbents owing to the COOH groups in their structure which promote the development of complexes with the targeted metal ions in water. Titanate nanotubes were produced in this work and utilized to create an extra G- nanocomposite that formed in titanium alginate (T/G). Few studies have focused on biopolymer-supported titanate nanotube composites, despite their potential advantages. Additionally, although numerous studies have investigated the removal of some heavy metals such as Cu²⁺, As³⁺, and Fe³⁺ ions from water, the research on the synergistic removal of Cs⁺ and Sr²⁺ remains limited. These gaps highlight the need for the present study, which aims to evaluate both the nanomaterials and their nanocomposites as effective adsorbents for Cs⁺ and Sr²⁺. So, the main aim of this study is to investigate the management of Cs^+^ and Sr^2+^ in synthetic wastewater by using TNTs and titanate alginate nanocomposite (T/G) beads. The second aim was to assess the removal efficiency of Cs^+^ and Sr^2+^ for the developed composite after the modification. Besides, the impact of the influencing factors: initial concentration, pH, contact time, and adsorbent weight on the synergistic removal performances of Cs^+^ and Sr^2+^ were discussed. Moreover, different non-linear adsorption isotherm modelling was used to evaluate the distribution of adsorbates onto TNTs in addition to five kinetics models with error analysis. Finally, for Cs^+^ and Sr^2+^, a comprehensive synergistic adsorption mechanism was proposed. Moreover, the reusability and regeneration potential of these adsorbents have been discussed to determine their practical applicability.

## Experimental work

### Materials and reagents

All chemicals were processed exactly as they received without any modifications. Crystalline anatase (TiO_2_) was provided from Loba Chemie laboratory reagents (India), while NaOH ACS grade and HCL were used to adjust the pH. The solutions were prepared in distilled water and millpore water. CaCl_2_·2H_2_O was provided from Oxford laboratory reagents Co. (India). The radioactive metals in the form of CsCl and SrNO_3_ were supplied from Merck (Germany). Na alginate with an average molecular weight 420 kDa was purchased from Oxford laboratory reagents (India).

### Preparation of titanate nanotubes (TNTs)

The hydrothermal technique was used to synthesize titanate nanotubes by dissolving 10 g of TiO_2_ powder and 200 g NaOH in 500 mL of distilled water at 1000 rpm for 37 min. After opening the autoclave, a milky solution was yielded and moved to a Teflon-lined stainless-steel autoclave (1 L). Then it was left in an oven for 16 h at 160 °C. The precipitate was then washed to remove NaOH. Water was added during stirring for 10 min, clear water was removed by filtration, washing was repeated many times, and the precipitate was then dried at 80 °C for 19 h^8^.

### Synthesis of alginate (G)

The ionic gelation route was applied to prepare the desired alginate microparticles^9^. Na alginate (aqueous solution of 3% w/v) slowly dropped into 0.1 L calcium chloride solution (2% w/v), and this process was performed under stirring at 300 rpm. After that, the developed microparticles were strengthened in calcium chloride solution overnight. Finally, the obtained alginate microparticles were collected, washed with distilled water, left to dry at 30 °C overnight, and kept in sealed vessels until additional studies were conducted.

### Synthesis of T/G nanocomposite

Titanate/alginate nanocomposite was prepared according to a previously reported process^10^. To prepare T/G nanocomposite, TNTs nanoparticles have been mixed with 3% w/v from a sodium alginate in an aqueous solution at a weight ratio of T: G = 1:3 for 3 h with strong stirring, following by a gradually dropping of this homogenous solution into CaCl_2_ (2% w/v). The nanocomposite was strengthened by keeping it in the CaCl_2_ solution overnight, followed by separation from the solution, and washing with distilled water and then dried at 30 °C overnight, and finally stored.

### Characterization of the TNTs, G and T/G nanocomposite

Alginate, TNTs and T/G nanocomposite were characterized by various analyses such as X-ray diffraction analysis (XRD), Field Emission Scanning Electron Microscopy with Energy Dispersive X-Ray Spectroscopy (FESEM-EDX), Fourier transmission infrared spectra (FTIR), zeta potential, N_2_ adsorption/desorption isotherms. The nanostructures were characterized by XRD patterns, and the data were used to analyze the crystal structure information obtained with a PANalytical (Empyrean) X-ray implying Cu Kα radiation at a wavelength 0.154 cm^−^^1^ in a scan range of 5.02° to 79.98°, at scan step 0.02° with accelerating voltage and current of 40 kV, and 35 mA, respectively. The morphology and mapping images were analyzed by FESEM-EDX micrographs of Quanta FEG 250 (Switzerland), at a 15 kV accelerated voltage. The surface characteristics were measured using N_2_ adsorption/desorption isotherms developed from BEL SORP MAX (Japan). Two methods; Brunauer Emmett–Teller isotherm (BET) and the Barrett–Joyner–Halenda (BJH) have been applied to calculate the specific surface area and pore size distribution, respectively. The surface functional groups were determined using FT-IR spectrometer (Bruker, Vertex 70). The materials were well mixed with KBr to form a pellet before being crushed. At 25 °C, the spectra have been detected at wave number ranging from 400 to 4000 cm^−^^1^.

### Cesium and strontium measurements

The metal ion concentration of Sr^+ 2^ has been measured by (ICP-OES) Inductively Coupled Plasma- Optical Emission Spectrometry (Perkin Elmer, model Optima 5300 DV, USA) at wavelength 228.2–232.2 nm, Gem cone nebulizer with normal cyclonic spray chamber with integration time from 0.01 to 5 s and pump flow 2 mL/min. The concentration of Cs^+^ was measured by Flame Atomic Emission spectrophotometer (Perkin Elmer, model an Analyst 100, USA) at wavelength 852.1 nm slit 0.2 using Gem tip nebulizer with integration time of 0.3 s and flame condition is air/acetylene.

### Metals uptake arrays

**Fig. 1:**
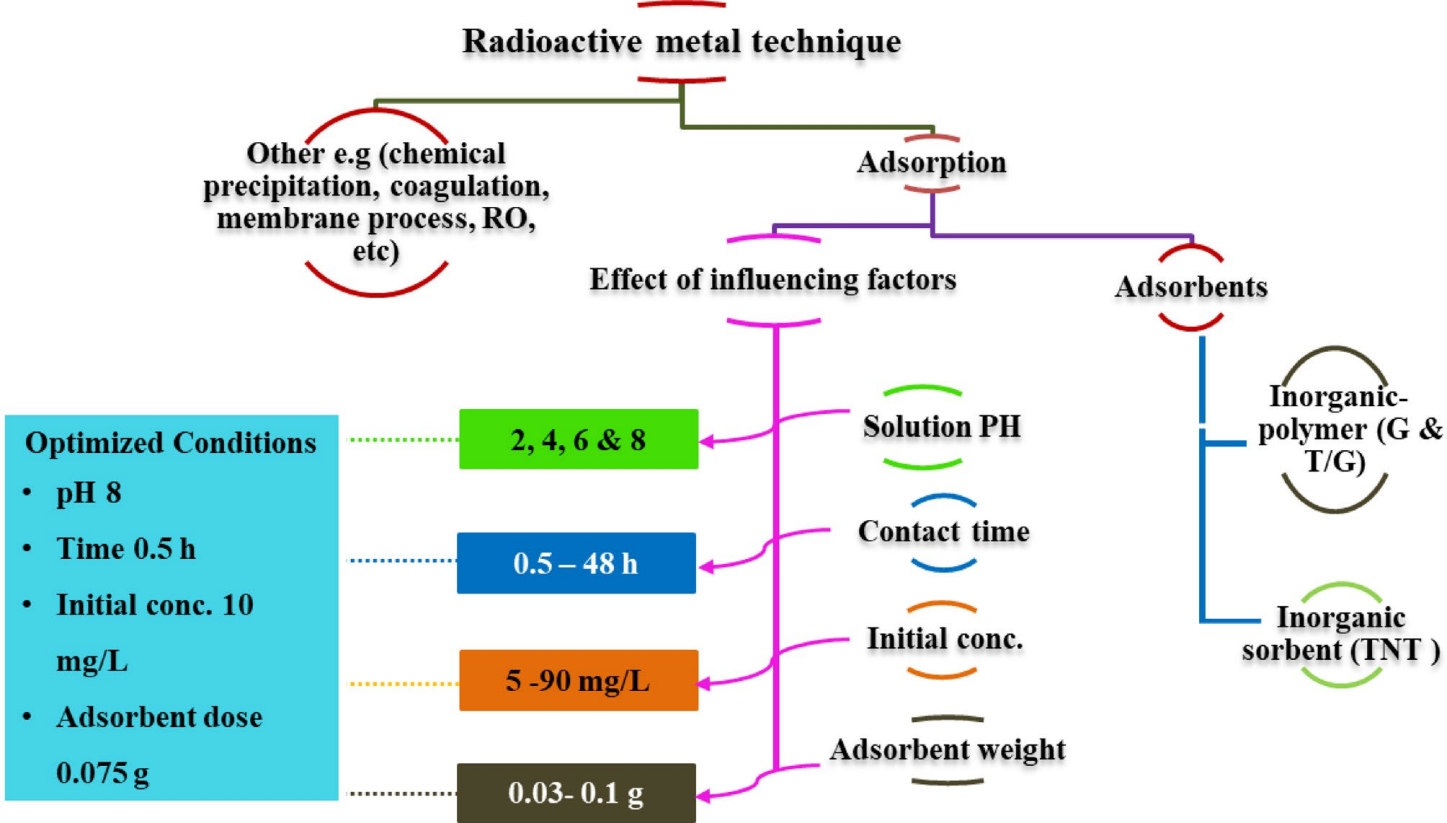
A schematic diagram listing the adsorbents, adsorption paramters affecting the removal of radioactive metals from the solution.

The ions of Cs^+^ and Sr^2+^ were removed using a batch adsorption process in aqueous solutions at room temperature with stirring (200 rpm). Many factors impacting the treatment of the radioactive elements in the solution have been optimized. To determine the adsorption capacity, the influencing operational parameters were optimized and concluded in Fig. 1. The amount of metal ions (q_e_) adsorbed at equilibrium was calculated according to Eq. (1).

1 $${\mathrm{q}}_{{\mathrm{e}}} = ({\mathrm{C}}_{{\mathrm{i}}} - {\mathrm{C}}_{{\mathrm{e}}} )*{\mathrm{V}}/{\mathrm{M}}$$

Which C_i_ and C_e_ (mg/L) are the initial and final concentrations of the metal ions, respectively. V (L) is the volume of the aqueous solution. M (g) is the mass of TNTs and T/G. The efficiency of the removal (R%) was calculated by Eq. (2).

2 $${\mathrm{R}}\% = (({\mathrm{C}}_{{\mathrm{i}}} - {\text{ C}}_{{\mathrm{e}}} )/{\mathrm{C}}_{{\mathrm{i}}} ) \times {\mathrm{1}}00\%$$

To guarantee dependable and reproducible adsorption results, the batch experiments were generally carried out in three repeated runs unless noted otherwise. The obtained measurements were summarized as mean values, and the adsorption capacities and removal percentages were derived from these averaged data. Applying this procedure follows common practice in adsorption research and reinforces the reliability of both the kinetic and isotherm interpretations.

### Regeneration and reusability

The reuse capability of adsorbents was investigated to determine their effectiveness over successive operational cycles. In case of T/G composed after the adsorption step was completed, the beads were collected and subjected to an initial rinsing stage. The particles were washed several times with deionized water (approximately ten-fold their volume per wash) under mild agitation to remove loosely attached ions and residual medium. The beads were then transferred into a dilute hydrochloric acid solution (0.1 M) and gently shaken for 60 min to release the previously adsorbed metal ions. The acidic extract was separated and retained for subsequent elemental analysis. Following desorption, the beads were repeatedly rinsed with deionized water until the pH of the washings approached neutrality (≈ 6–7), ensuring that all acidic residues were removed and that the material was suitable for subsequent re-crosslinking. To restore the structural integrity of the alginate matrix, the cleaned beads were immersed in a calcium chloride solution (2% w/v). The presence of Ca²⁺ ions promote the re-establishment of ionic junction zones within the alginate chains, reinforcing the hydrogel framework. The beads remained in the CaCl₂ solution for 20–30 min and were then rinsed twice with deionized water to eliminate excess calcium. In cases where the alginate beads had been left unused for an extended period and had undergone noticeable shrinkage or dehydration, a rehydration step was performed prior to regeneration. The dried beads were soaked in deionized water for an extended period (overnight) under gentle agitation to allow gradual water uptake and swelling. Once the beads regained their hydrated form, they were subjected to the same CaCl₂ reactivation procedure to rebuild the ionic crosslinks necessary for mechanical stability. This rehydration–reactivation sequence restored the beads to a functional state suitable for further adsorption cycles. However, re-use of the beads was not attempted under certain conditions. Beads that exhibited severe fragmentation, irreversible loss of mechanical integrity, excessive microbial growth, or a decline in structural cohesion after rehydration were considered unsuitable for further processing.

### Adsorption isotherm modeling

Adsorption isotherms are important in giving information about the interactions among the adsorbate/adsorbent, and the adsorption mechanism. Hence, eleven isotherm models have been studied. The adsorption capacities of the adsorbent at equilibrium for Cs^+^ and Sr^2+^ were analyzed corresponding to Langmuir (Eq. S1), Freundlich (Eq. S3), Langmuir-Freundlich (Eq. S4), Temkin (Eq. S5), Dubinin–Radushkevich (Eqs. S6 and S7), Sips (Eq. S8), Redlich-Peterson (Eqs. S9 and S10), Toth (Eq. S11), Kahn (Eq. S12), Baudu (Eq. S13) and Fritz Schlünder (Eq. S14) models^11^. The adsorption isotherm experiments for Cs^+^ and Sr^2+^ occurred at initial concentrations ranging from 5 to 1140 mg/L at 25 °C for Sr^2+^ and from 5 to 530 mg/L for Cs^+^ at 20 mL of solution, pH 8, and adsorbent mass 0.075 g for 2 h. After adsorption, the equilibrium adsorption amount was determined by the remaining concentration of Cs^+^ and Sr^2+^ in the supernatant, which was fitted to the eleven isothermal adsorption models.

### Kinetics studies

One of the most principal factors determining the rate of adsorbate removal and the adsorption effectiveness of an adsorbent is its adsorption kinetics. It is then used to optimize and design the actual industrial applications. Commonly, adsorption occurs in several steps: (1) adsorbate migration from the bulk solution to the surface of the adsorbent; (2) adsorbate diffusion into the pore interior and/or mobility along the adsorbent’s surface; and (3) adsorption occurs between the adsorbate and the accessible active sites. Anyone of these phases, or a mix of them, may most likely develop to the rate-controlling step of adsorption. Several kinetic models are available to recognize the complex dynamics of the process of adsorption. The fitting of the model is chosen depending on how near the correlation coefficient (R^2^) is to one in addition to the error calculation. The rate of adsorption of Cs^+^ and Sr^+2^ onto adsorbent has been studied using five kinetic models: pseudo first order (PFO) and pseudo second order (PSO), mixed first and second order (MFSO), Avrami and intraparticle diffusion (I-P) models according to Eqs. S15-S19, respectively. Both PFO and PSO models include all adsorption processes, involving external film diffusion, adsorption and intraparticle diffusion.

### Error analysis of isotherm and kinetic models

To ensure the reliability and accuracy of the non-linear isotherm modelling applied in this study, a comprehensive error analysis was carried out. Although R² is commonly used to evaluate model fit, it is insufficient on its own for non-linear regression. Therefore, additional statistical error functions including Sum of Squared Errors (SSE), Chi-square (χ²), Average Relative Error (ARE), and Root Mean Square Error (RMSE) were calculated according to Eq. S20–S23. These error functions allow for more accurate judgement of which model best describes the adsorption mechanism of Cs⁺ and Sr²⁺ onto T/G composites. The model showing the lowest values of SSE, χ², ARE, and RMSE, along with the highest R², was considered as the most suitable.

## Results and discussion

### Characterization analyses

The following sections discuss in more detail the characteristic features of the developed adsorbents.

#### Diffraction of X-ray

**Fig. 2:**
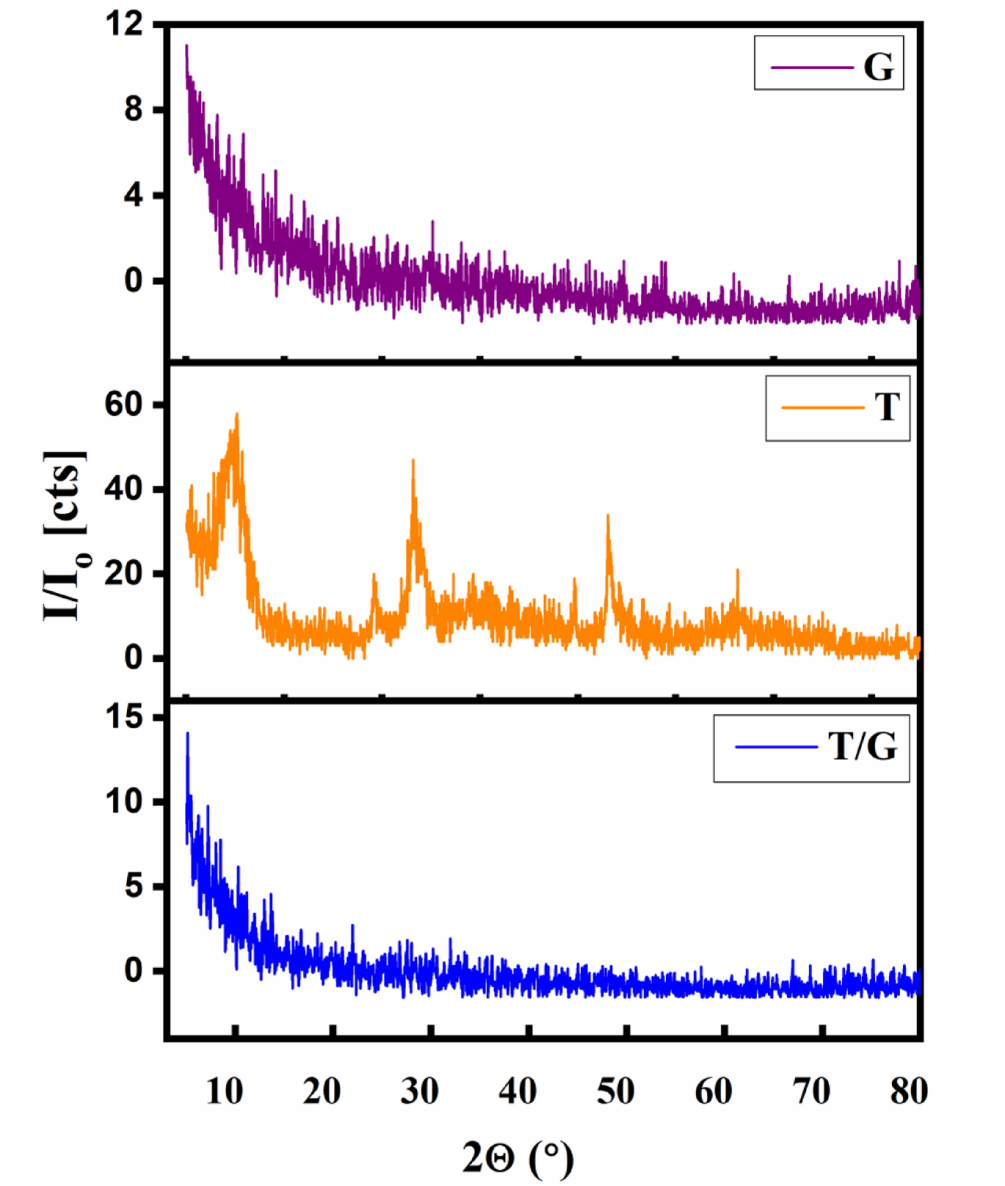
The XRD patterns of TNTs, G, and T/G.

The diffraction profiles obtained for TNTs, G and T/G composite are presented in Fig. 2. The reflections appear at (9.66 ^o^, 24.34 ^o^, 28.18^o^, 48.22^o^ and 60.86 ^o^ are consistent with the crystalline framework of tubular titanate (Na_2_Ti_3_O_7_) listed under [ICDD card no. 31-1329] and assigned to the P21/m space group. The crystallite dimensions were evaluated using Scherrer’s equation (Eq. 3).

3 $${\mathrm{D}}\, = \,0.{\mathrm{94}}\lambda /\left( {\beta {\mathrm{cos}}\theta } \right)$$

where λ denotes X-ray target wavelength, D is the crystallite size, β is corrected full width at half maximum of the diffraction peak, and θ corresponds to the Bragg angle. Using this approach, the value of crystallite size is 11.4 nm for TNTs. After radionuclide adsorption, the main structural peaks remained intact, confirming that the overall crystal framework of TNTs was preserved. Minor peak broadening or intensity variation was detected, which may be linked to a partial ion exchange within the titanate layers and slight distortion associated with metal incorporation. The absence of distinct diffraction peaks for the T/G nanocomposite indicates that the incorporated TNTs are present in a very small proportion relative to the amorphous G matrix. This observation also suggests that TNTs are homogeneously dispersed throughout the polymer network, which enhances the shielding capability of the composite.

#### Scanning electron microscopy

**Fig. 3:**
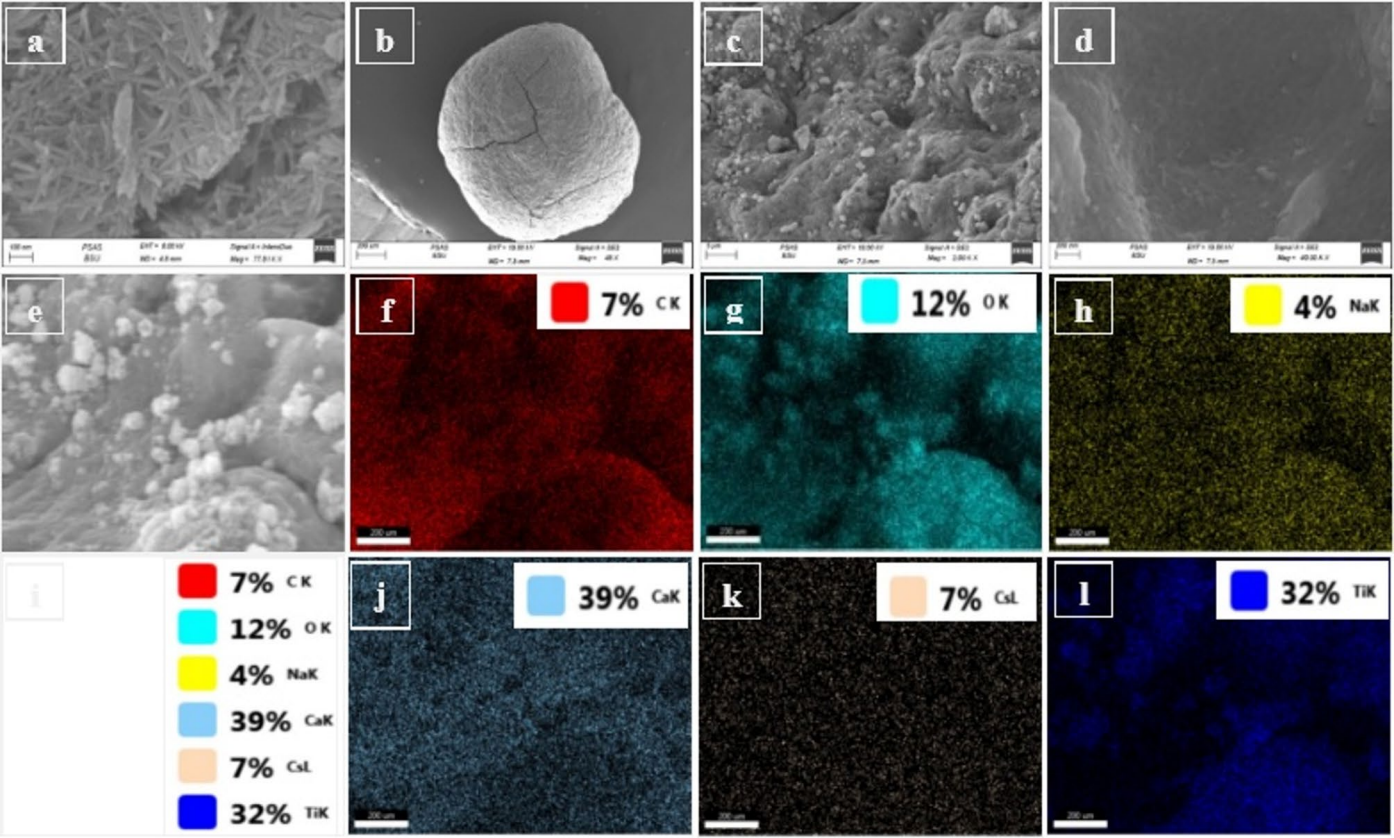
FESEM micrographs of TNTs (**a**), Cs@T/G (**b–d**) and EDX-mapping of Cs@T/G (**e–l**).

Figure 3a depicts the shape of TNTs nanotubes. The micrograph obviously showed that TNTs are in the form of tubes, with an average diameter of 10 nm. The nanotubes are present as aggregates and their distribution is non-uniform. Titanium nanotubes were formed via the hydrothermal reaction of concentrated NaOH solution with anatase at 160 °C. The crystalline structure of TiO_2_ polymorphs is anatase. It is represented using typical TiO_6_ octahedra that share vertices edges to build up a 3-dimensional oxide structure. It is possible that part of the raw materials’ Ti-O-Ti bonds break when they react through an alkaline solution, hence forming layered titanates consist of octahedral units of TiO_6_ with alkali metal ions in the form of thin tiny sheets. According to one of the reported proposed mechanisms, the titanate sheets undergo exfoliation into one- or double-layered nanosheets during autoclaving. These nanosheets then rolled to nanotubes with a gradual growth rate, presumably because of the concentrated of NaOH solution^3^. FESEM results demonstrate an oblate shape of the alginate microparticles characteristic with an average grain size about 1200 μm. It appeared a thick brain-like shape with obvious porosity and lower surface area. The morphology of T/G composite after adsorption of Cs^+^ was investigated and illustrated in Fig. 3b–d. It can be noticed the development of thick layer onto the surface of nanocomposite. This thick layer may refer to the development of a multilayer chemosorption (Fig. 3c). On the other hand, Fig. 3d suggests that physisorption also coexists where there is a uniform layer on the surface of the nanocomposite (i.e., the surface became more homogeneous). The FESEM-EDX-mapping images of T/G nanocomposites are illustrated in Fig. 3(e–l) indicate uniform distribution of TNTs in the G matrix and show the relatively porous surface of T/G nanocomposites as well as the uniform distribution of Cs^+^ after adsorption over G and TNTs nanotubes surfaces.

#### FT-IR spectrometry

**Fig. 4:**
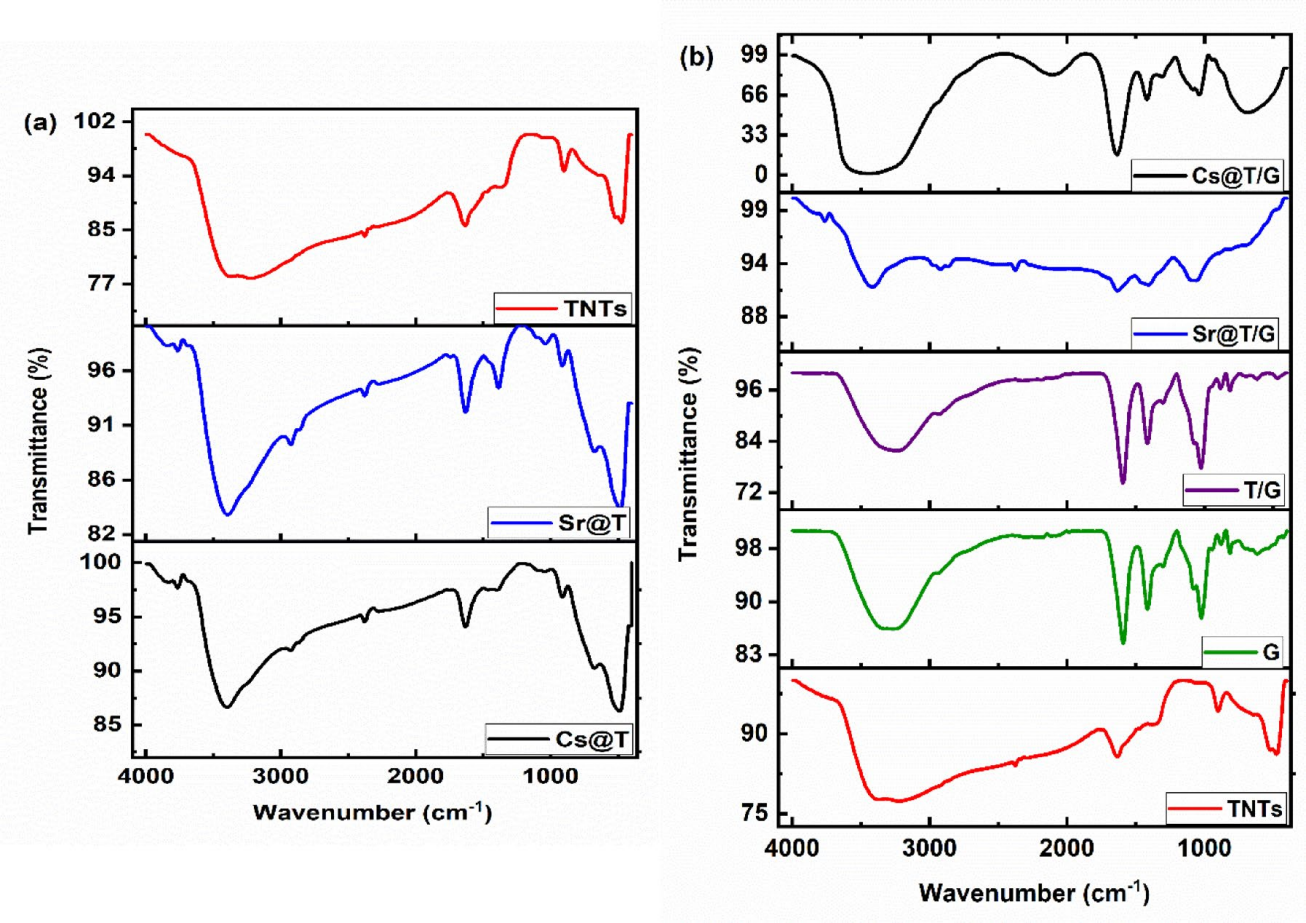
FTIR spestra of TNTs, Cs@T, and Sr@T (**a**), and G, TNTs, T/G, Sr@T/G and Cs@T/G (**b**).

The analytical technique of FT-IR provides information on the adsorption mechanism because it gives information on the molecular interactions and changes that occur throughout the adsorption process. The resultant spectrum is a unique fingerprint of the molecular composition of the material. FT-IR was employed to characterize TNTs, Cs@T, Sr@T, (Fig. 4a) and TNTs, G, T/G, Cs@T/G, Sr@T/G (Fig. 4b). FT-IR spectrum of TNT is shown on Fig. 4a. The broad band appeared at 3400–3200 cm^− 1^ and the peak observed at 1633 cm^− 1^ could be described as the stretching and bending vibrations of -OH and H-O-H, respectively^12^. The peak observed in 475–520 cm^−1^ is associated to the stretching vibration of Ti-O^13^. The carbonate band in TNTs was appeared at 1382 cm^−1^. The band at ~ 1070 cm^−1^ originated from the NO_3_ group^14^. Hence, TNTs displayed characteristic bands corresponding to –OH stretching vibrations, surface-bound water, Ti–O lattice modes, and carbonate-related bands originating from atmospheric CO₂ interaction. Following metal uptake, noticeable shifts were observed in several key vibrational regions. After adsorption of Cs^+^ and Sr^2+^, the broad –OH stretching band exhibited a slight displacement and transmittance increment, indicating the involvement of surface hydroxyl groups through hydrogen bonding or direct metal–oxygen interaction. The vibration bands at ~ 500 cm^−1^ shift towards a higher wave number side, and it was positioned at around 489–675 cm^−1^ after the adsorption of Sr^2+^/Cs^+^. It is indicating that the shift was detected when Cs^+^ and Sr^2+^ are adsorped onto TNTs^15^. For Cs@T and Sr@T, the carbonate band was appeared at 1398 and 1386 cm^−1^, respectively. After Sr^2+^/ Cs^+^ uptake, the shift was combined with increment in the intensity of carbonate band.

 The spectrum of G (Fig. 4b) shows the characteristic peaks of G. The peak observed around 3271 cm^− 1^ is appointed to the stretching vibration of -OH. The spectral transmissions observed at 1416 and 1591 cm^− 1^ indicate the symmetric and asymmetric carboxylate bond stretching vibrations, respectively^8^. The band peaks at 1079 cm^− 1^ and 1022 cm^− 1^, that correspond to the stretching vibrations of the OC–OH bond of the glycosidic band linkage and C = C^16^. It is obvious from Fig. 4b that T/G gave nearly same spectrum of G this might be due to the little amount of TNTs incorporated in the G matrix. The spectrum exhibits the bands of the alginate’s -OH and -COO groups, as well as the Ti-O vibrational bands. Furthermore, as compared to the original spectra of G and TNTs, the intensity of the peak changes with a little shift in frequency. O-H stretching band at 3271 cm^− 1^ in G is also visible and shifted to 3237 cm^− 1^ in T/G. The band observed in both G and T/G at 1079 and 1072 cm^− 1^ respectively, can be attributable to O–C asymmetric flexible vibration. The absorption peak at 2931 cm^− 1^ was attributed to the intramolecular C–H vibrations. These changes in addition to the appearance of new peaks at 470 and 696 cm^− 1^ might be due to the interaction of TNTs with the alginate biopolymer, demonstrating that the T/G preparation was successful. According to (Fig. 4b), the spectra of the T/G after sorption of Cs@T/G and Sr@T/G show a shift in the absorption bands and deformation of –OH, C–H vibrations, –COO–, and C–O–C groups developed at 3440 and 3417 cm^− 1^, 2928 and 2868 cm^− 1^, 1637 and 1630 cm^− 1^ and 1085 and 1060 cm^[− 1 17^, respectively because of the electrostatic interaction^18^. The asymmetric and symmetric COO⁻ vibrations also showed measurable shifts, suggesting participation of carboxylate-like groups in coordinating or electrostatically interacting with Cs⁺ and Sr²⁺. Furthermore, the Ti–O lattice band experienced a minor shift, supporting the ion-exchange mechanism in which Na⁺ ions within the titanate layers are partially replaced by the incoming radionuclides. Disappearance of the peaks at 470 and 696 cm⁻¹ in the Sr@T/G spectrum indicates direct interaction of Sr²⁺ with titanate lattice vibrations, suggesting partial distortion or replacement of Na⁺ within the Ti–O framework, while the peak at 489 cm^− 1^ become less intense with Cs⁺ adsorption. The decreased intensity of the ~ 489 cm⁻¹ band in Cs@T/G reflects stronger Cs⁺–Ti–O interactions, confirming the contribution of ion-exchange and surface complexation mechanism which is agreed with a previous work^19^. These spectral changes (appearance, disappearance, shifting and change in intensity) collectively confirm the multi-mechanistic adsorption pathway involving electrostatic attraction, surface complexation, and ion exchange.

#### N_2_ adsorption/desorption isotherms

**Fig. 5:**
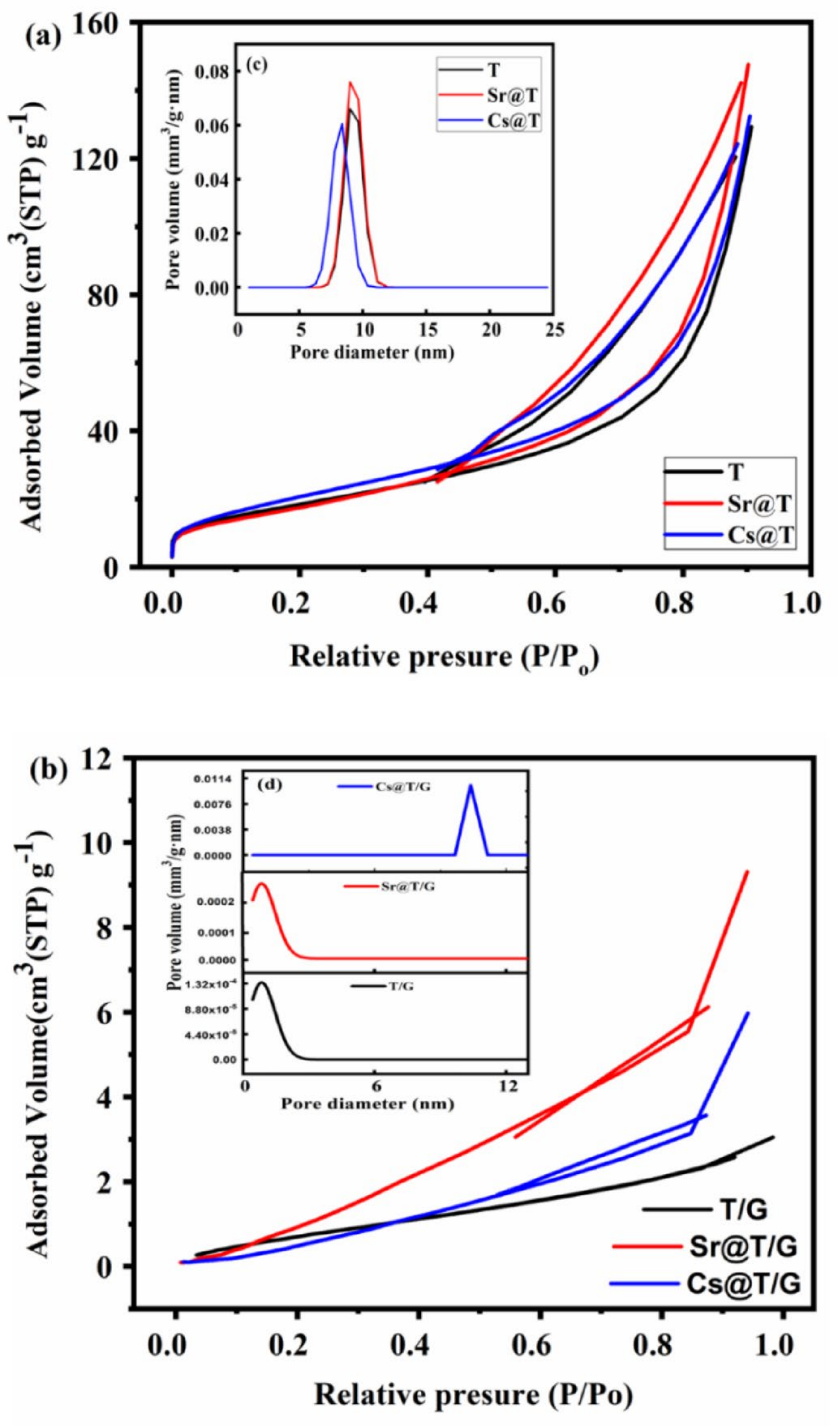
Nitrogen adsorption/desorption isotherms and pore size of TNTs (**a**, **c**) and T/G (**b**, **d**) before and after adsorption by Cs^+^ and Sr^2+^, respectively.

The specific surface area and pore size distribution were established using BET and BJH methods developed from N_2_ adsorption/desorption isotherms for TNTs, Cs@T and Sr@T (Fig. 5a) and T/G, Cs@T/G and Sr@T/G (Fig. 5b). The pristine nanotubes exhibited a large surface area (71.59 m^2^/g) and mesoporous structure (Fig. 5c) which is favourable for hosting the solvated ions. It can be seen that after Cs⁺ and Sr²⁺ adsorption, the BET specific surface areas increased relatively. This may be attributed to the development of new functional groups resulting from the intraction of Cs^+^ and Sr^2+^ and TNTs that may attract more nitrogen molecules resulting in a realtive increase in the specific surface area which is agreed with the morphological results (Fig. 3c). The adsorption and desorption isotherms of N_2_ after adsorption were reliable with type IV isotherms on the IUPAC classifications and the noticed hysteresis loops (of type H3) at high P/P_o_ show that the materials are mesoporous^20^. Following the adsorption of Cs^+^ onto the nanocmposite, it can be noticed that the pore size distribution was signifcantly change (Fig. 5d). However, the distrbution of the pore size depends on the intensity of the interaction between the solid surface and the adsorbate as well as the nature of adsorption; chemisorption or/and physisorption^21^. For T/G nanocomposite, this behaviour may be attributed to the improved pore accessibility resulting from ion-induced rearrangement of surface hydroxyl networks or/and ion exchange between cations; Na^+^ and Cs^+^ resulting in a significant change in the T/G matrix after Cs adsorption.

### Metal uptake study

The adsorption decontamination of wastewater is usually influenced by several factors that must be optimized for a better adsorption process. The sections that follow discuss in more detail the effect of solution pH, Cs^+^ and Sr^2+^ initial concentrations, contact time, sorbent dosage, on the adsorption of Cs^+^ and Sr^2+^ onto the developed adsorbents.

#### The effect of pH

**Fig. 6:**
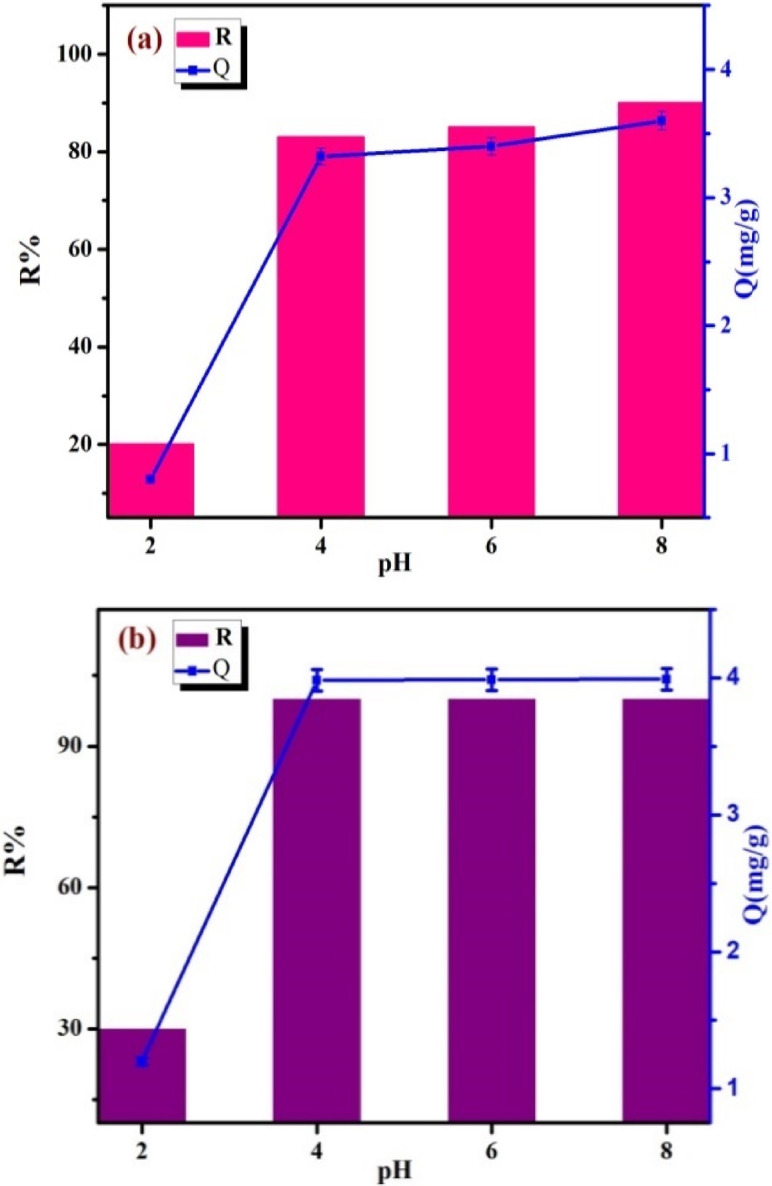
The effect of solution pH on the removal efficiency of Cs^+^ (**a**) and Sr^2+^ (**b**) ions by T in range of pH 2.0–8.0 using 20 mL of Cs^+^ (10 mg/ L) and Sr^2+^ (10 mg/ L), 0.05 g of TNTs, for 2 h.

The pH of the solution impacts the surface charge of adsorbents in suspension, which has an immediate influence on the electrostatic interaction between adsorbents and adsorbates. Figure 6 demonstrates that raising the pH of the solution enhances the adsorption efficiency significantly. Because Cs^+^ and Sr^2+^ ions increase in water as cations, it is easy to predict that a negatively charged surface aids the adsorption of Sr^2+^ and Cs^[+ 22^. The point of zero charges (pH_pzc_) is 3.3 for TNTs. This is describes the sudden increase in both metals’ adsorption capacities and removal efficiencies on the sorbent after this value^23^. This is also indicates that Na^+^ can compete with Sr^2+^ and Cs^+^ for ion exchange on TNTs while also affecting electrostatic behavior^22^. When pH range is between 2.0 and 3.0, as show in Fig. (6 a& b), the adsorption capacity was very low due to the electrostatic repulsion between the TNTs and Cs^+^ and Sr^2+^ causing a huge surface repulsion because the surface of TNTs was surrounded by many H^+^ ions, which prevented the adsorption of Cs^+^ and Sr^2+^. In contrast, when pH > pHpzc (3.3), the increasing negative charges on TNTs surface led to an increase of numerous adsorption sites. The adsorption capacity as well as the efficiency of the removal of TNTs towards Sr^+ 2^ is greatly higher than Cs^+^ showing that the selectivity of TNTs is more for Sr^+ 2^ than Cs^+^.

#### Impact of contact time

**Fig. 7:**
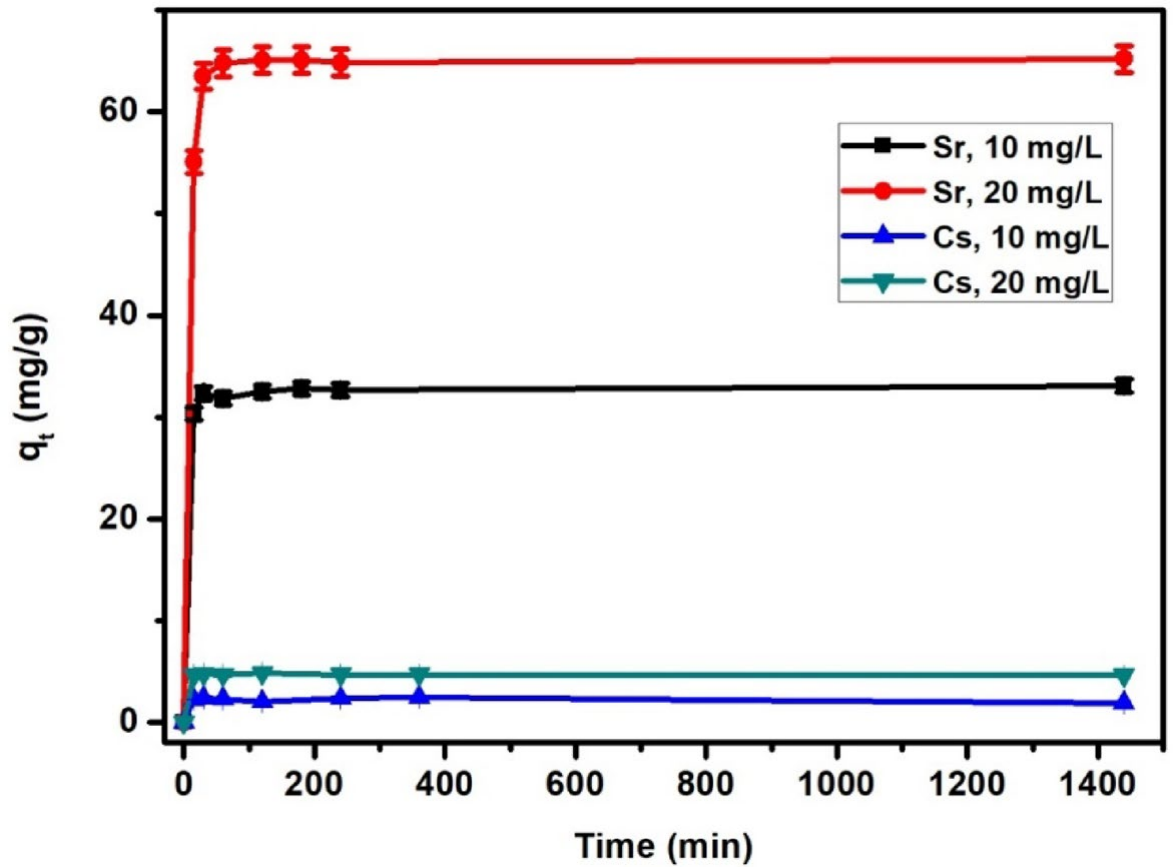
Impact of time on the adsorption of Cs^+^ and Sr^2+^ onto TNTs using Sr^2+^ (10 and 20 mg/ L) and Cs^+^ (10 and 20 mg/ L) at pH 8, volume 250 mL.

The impact of residence time on the adsorption capacity of Cs^+^ and Sr^2+^ onto TNTs at the optimum conditions is shown in Fig. 7, the residence time raised from 0 to 15 min, the adsorption capacities of both metals increase to 2.26 mg/g and 4.65 mg/g for Cs^+^ of 10 and 20 mg/L, respectively and to 30.33 mg/g and 55.07 mg/g for Sr^2+^ of 10 and 20 mg/L, respectively, until a specific time (30 min) and an equilibrium stage after which no more increase was recorded. This is because the surface of TNTs has many free sites that are available for Cs^+^ and Sr^2+^ to interact and bind to during early contact time. With a longer contact time, the adsorption capacity (q_e_) becomes constant because of saturation of the adsorbent’s active sites, beyond which no further change is predicted. Thus, the adsorption achieved in two phases: an initial quick phase followed by a second slower phase until equilibrium was reached.

#### Impact of adsorbent mass

**Fig. 8:**
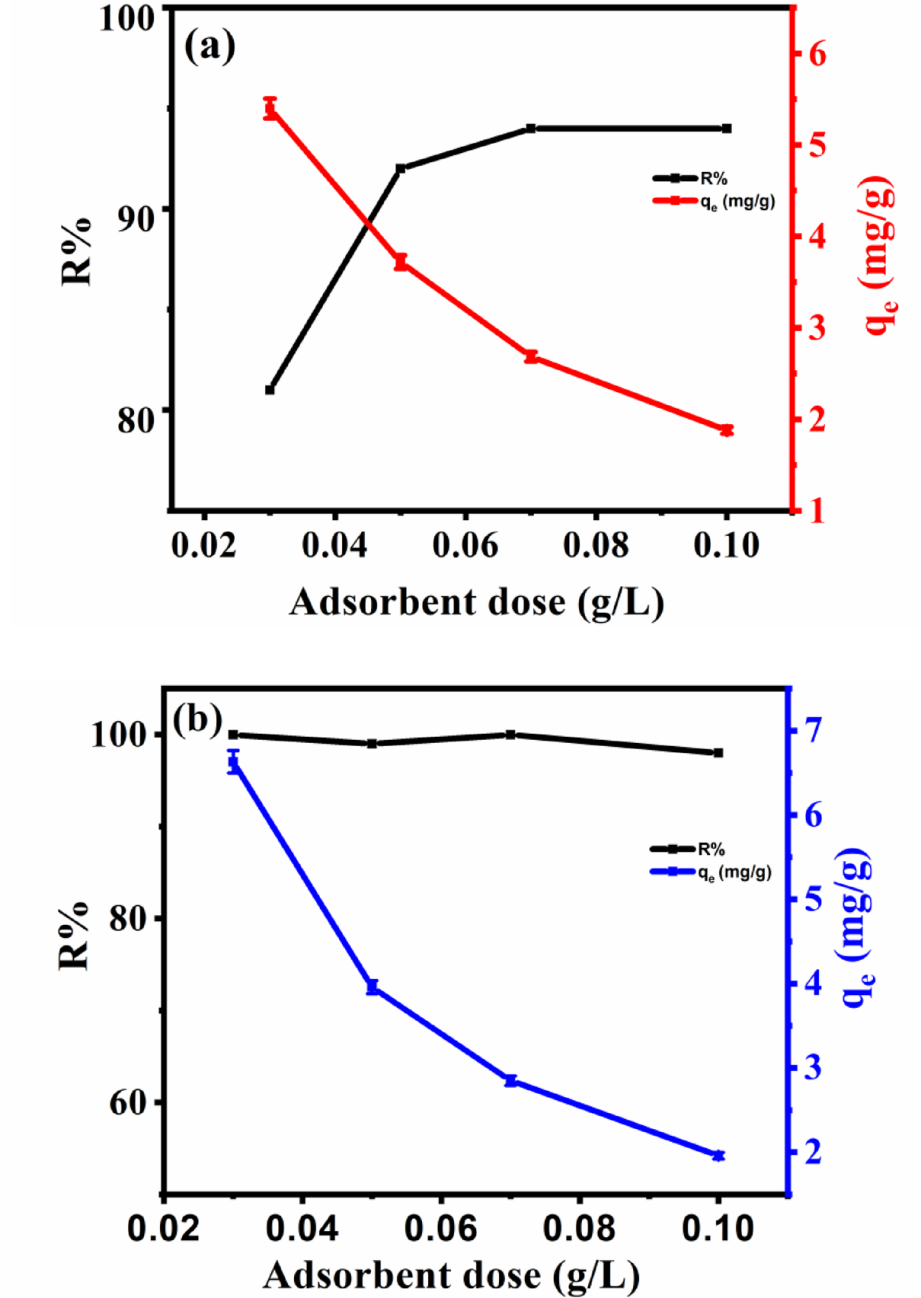
Effect of adsorbent mass of TNTs (0.03 to 0.1 g/L) on the adsorption and removal efficiency of Cs^+^ (**a**) and Sr^+ 2^ (**b**) at pH 8 using 20 mL of 10 mg/L of adsorbate (Cs^+^ and Sr^2+^) for 2 h.

To investigate the impact of adsorbent dosage, various amounts of TNT concentrations ranged from 0.03 to 0.1 g/L were mixed with metal ions, as can be seen in Fig. 8. Adsorption capability of Cs^+^ (Fig. 8a) and Sr^2+^ (Fig. 8b) are inversely related to adsorbent dosage. The adsorption capacity of Cs^+^ decreased from 5.4 mg/g to 1.8 mg/g (Fig. 8a) and Sr^2+^ from 6.6 mg/L to 1.96 mg/L (Fig.8b) when TNTs dosage increases from 0.03 g/L to 0.1 g/L. The reduction in the adsorption of the metals from aqueous solution with increasing TNTs dosage is due to particle aggregation, which reduces the diffusion path length^24^. Figure8a additionally represents the effect of adsorbent dose on the removal performance of Cs^+^ from water. It was found that increasing the adsorbent dose from 0.03 g to 0.1 g increased the removal efficiency. This is due to the presence of more active adsorption sites and an increase in the active surface area of nanoadsorbents. Figure 8b demonstrates that the removal efficiency of Sr^2+^ from water is not significantly affected by the variation of the dose of TNTs owing to the high selectivity of TNTs towards Sr (around 100%) which agreed with pH results.

#### Effect of initial concentration of Cs^+^ and Sr^2+^

**Fig. 9:**
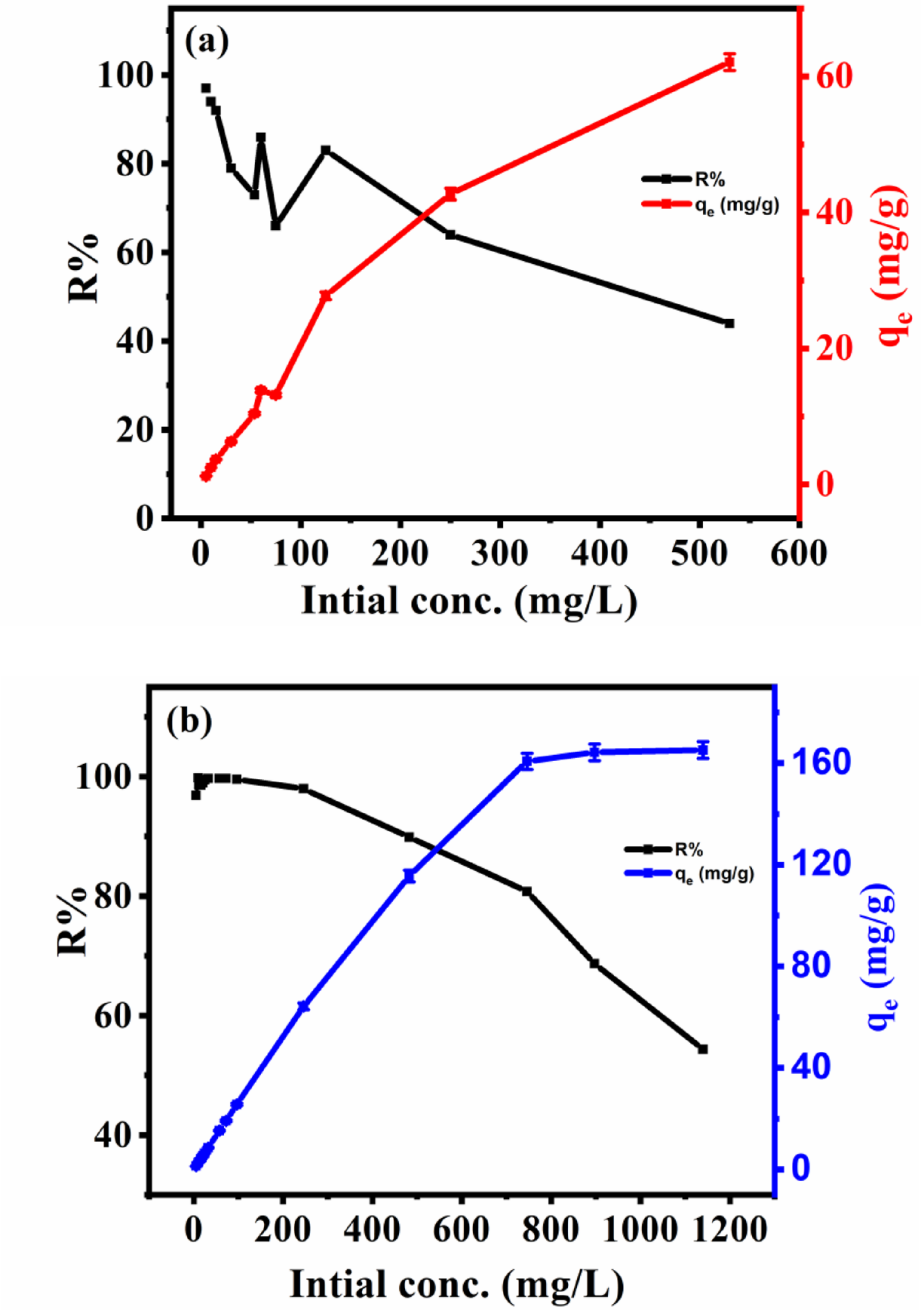
Effect of initial concentrations for Cs^+^ (**a**) and Sr^2+^ (**b**) in 20 ml of solution at pH 8 using 0.075 g of TNTs, for 2 h.

The removal efficiency is related directly to the initial metal ion concentration. This might be due to an increase in driving force from the concentration gradient according to the mass transfer. The change in adsorption capacity with initial Cs^+^ and Sr^2+^ concentration (5 mg/L to 500 mg/L) and (5 mg/L to 1140 mg/L), respectively under optimal condition is illustrated in Fig. 9. The results illustrate that adsorption capacities sharply increased with increasing Cs^+^ and Sr^2+^ concentrations, owing to higher mass transfer driving forces causing increased interaction between Cs^+^ and Sr^2+^ molecules at higher concentrations. Figure 9 is also indicates that the removal efficiencies of the metals ions decreased with increasing the metals initial concentrations due to the availability of more vacancies onto the surface of TNTs that are not fully filled at lower concenrations/loading of the metals.

### Adsorption isotherm modeling

**Fig. 10:**
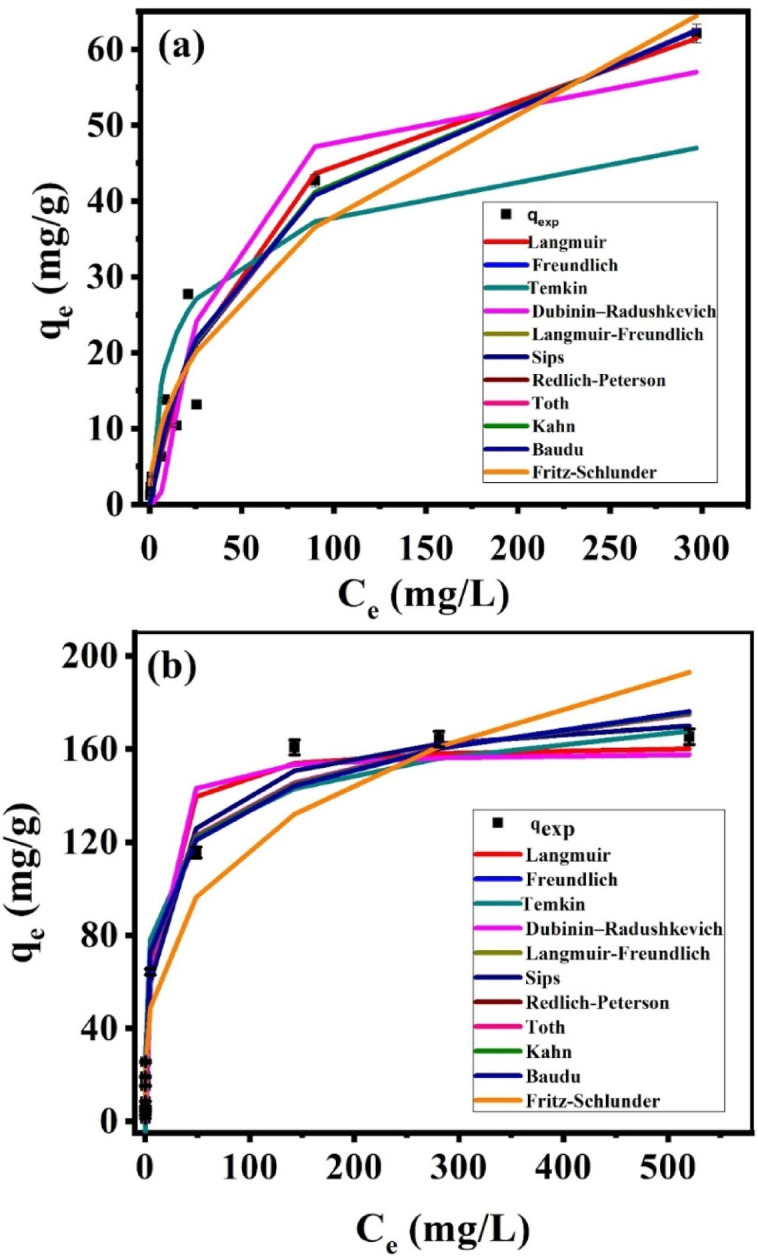
The adsorption isotherm modeling of the adsorption of Cs^+^ (**a**) and Sr^2+^ (**b**) at pH ~ 8, 0.075 g of adsorbent, contact time 2 h and volume 20 mL at 25 °C.

Nonlinear regression was used to determine the parameters of the isotherms, utilizing the solver addition with the Microsoft Excel spreadsheet for calculations. This process gives an exact mathematical calculation for isotherm parameters utilizing the first original form of the isotherm equation. Figure 10 illustrates the fitting of eleven models (Langmuir, Freundlich, Temkin, Toth, Dubinin-Radushkevich, Langmuir-Freundlich, Redlich-Peterson, Kahn, Baudu, Sips and Fritz-Schlünder) to the experimental data for the correlation between q_e_ and C_e_. The fitting is based on the q_m_ and q_exp_ results, as well as comparing the values of R^2^. Table 1 shows the suitable fitting correlation coefficients (R^2^) and the equilibrium constants. Langmuir-Freundlich and sips models (Fig. 10a) are the best to describe the data of Cs@T adsorption system where R^2^ values are high along with an agreement between the calculated values and the experimental one. Figure 10a is also show that the adsorption system Cs@T is fitted by D-R model with a theoretical q_m_ 61.9 mg/g very close to q_exp_ (62.1), in addition to a sufficient correlation coefficient value (R^2^ = 0.89). This model helps describe the formation of multilayers on microporous adsorbents. Because it can’t assume a homogeneous surface, which is more general than the L isotherm model. It can, however, be utilized to explain the heterogeneity of the adsorbent surface at low coverage. It is only applicable for intermediate adsorbate concentrations. It suggests that the adsorption procedure is controlled by a pore-filling mechanism. This is used to distinguish between chemisorption and physisorption. From Eq. (S7), the mean free energy value equals (E = 10 kJ /mol) and falls within range 8 < E < 16 kJ /mol, indicating that the procedure involves chemisorption. Freundlich model depends on the development of multilayer adsorbate across a heterogeneous adsorbent surface. Furthermore, the active sites showed varying in adsorption energies^25^. The correlation coefficient value (R^2^) = 0.94. The K_f_ values were determined to be 4.35, with the low K_F_ value which is associated with a short time (just 30 min) to reach equilibrium^26^. The slope (1/n_f_ = 0.47), it falls within the range 0 < 1/ n_f_ < 1. This indicates a good adsorption process; As a result, this model also fits the adsorption isotherm data. Although Langmuir model yields high R^2^ (0.94), the calculated q_m_ (74.6) is higher than the experimental one (q_exp_ = 62.1 mg/g). The separation factor calculated according to Eq. S2, (R_L_), = 0.9 at q_max_ is in range (0 < R_L_ < 1) which make the model could also describe the adsorption system Cs@T. The R-P model is suitable (R^2^ = 0.95) on the other hands, Temkin model is unsuitable for illustrating the adsorption system Cs@T where R^2^ is low (0.77).

The results demonstrated that the Sr@T adsorption system is well fitted by Langmuir-Freundlich and sips models (Fig. 10b) where R_2_ values are high in addition to that the experimental values are close to the calculated one. Langmuir model shows a high value of R^2^ (0.98), the k_L_ values were found to be 0.12 L/mg, the theoretical q_m_ (162.592 mg/g) is approached to q_exp_ (165.33 mg/g) and separation factor is in range (0 < R_L_ = 0.008 < 1). This suggests that the adsorption process occurs in monolayer adsorption at maximum adsorption with consistent adsorption distribution and all adsorption sites are equivalent, and each site is able to accommodate one molecule, there are no phase transitions, affinities over the homogeneous surface, and adsorbed molecules do not interact^11^. Freundlich model showed high R^2^ (0.95), The K_f_ values which were found to be 30.86 is due to the fast time (only 30 min) to reach equilibrium^26^ and the slope (1/n_f_ = 0.29) varying from 0 to 1 represents the adsorption intensity or surface heterogeneity, which showed that the adsorption of Sr^2+^ on the TNTs was favorable. The D-R model could also be applied to describe the Sr@T adsorption system with R^2^ = 0.96 and an agreement between the calculated and the experimental adsorption capacities. From Eq. (S7) the mean free energy value is less than 8 kJ /mol, suggesting a physisorption process (E = 7 kJ /mol). The experimental data were also well fitted by Temkin, R-P and Toth models with high R^2^ (0.97, 0.99 and 0.95 respectively). On the other hand, Kahn, Baudu and F-S models are less fitting to describe Sr@T and Cs@T system where the corresponding theoretical adsorption capacities of these models (75.39, 68.33 and 44.21 mg/g, respectively for Sr@T and 37.4, 13.02 and 16.8 mg/g, respectively for Cs@T are less than q_exp_ (162.59 and 62.1 mg/g) even with high R^2^ values resulted from these models (Table 1). In general adsorption is a mixed process that does not follow ideal monolayer adsorption; it might be a combination of physical and chemical (ion exchanging) adsorption, which is evidenced by pH data and characterization results.

**Table 1:** The values of the parameters of the adsorption isotherm models for Cs^+^ and Sr^2+^ uptake onto TNT.

Adsorption models		Parameter	Cs^+^	Sr^2+^
Two parameters isotherm	Langmuir	q_m_	74.67	162.59
		K_L_	0.02	0.12
		R^2^	0.94	0.98
	Freundlich	K_f_	4.35	30.86
		1/n_F_	0.47	0.29
		R^2^	0.94	0.95
	Dubinin-Radushkevich	q_m_	61.92	159.19
		K_ad_	0.005	0.001
		R^2^	0.89	0.96
	Temkin	b_T_	307.95	130.25
		A_T_	1.12	12.27
		R^2^	0.77	0.97
Three parameters isotherm	Redlich-Peterson	K_R_	1.62	56.95
		a_R_	0.07	0.74
		β	0.81	0.87
		R^2^	0.95	0.99
	Sips	q_m_	95.54	189.29
		Ks	0.02	0.17
		1/n	0.77	0.63
		R^2^	0.95	0.99
	Langmuir-Freundlich	_MLF_	95.6	189.29
		_LF_	0.008	0.06
		_LF_	0.77	0.63
		R^2^	0.95	0.99
	Toth	K_e_	1.31	49.37
		K_L_	0.07	0.74
		n	0.81	0.87
		R^2^	0.95	0.99
	Kahn	q_m_	37.41	75.39
		b_K_	0.04	0.7
		a_K_	0.76	0.86
		R^2^	0.95	0.99
Four-parameters isotherm	Baudu	q_m_	13.02	68.33
		b_0_	0.08	1.11
		x	0.28	0.15
		y	0	0
		R^2^	0.94	0.99
Five-parameters isotherm	Fritz-Schlünder	q_mFSS_	16.8	44.21
		K_1_	0.27	0.7
		K_2_	0.02	0
		m_1_	0.47	0.29
		m_2_	0	0
		R^2^	0.94	0.95

Based on the error analysis conducted in this study (Table S1); For Cs⁺ adsorption, Langmuir-Freundlich and Sips models showed the lowest SSE and χ², ARE, and RMSE values. For Sr²⁺ adsorption, Langmuir-Freundlich and Sips model demonstrated the lowest SSE and χ², and RMSE values, indicating heterogeneous surface interactions consistent with the structure of TNTs. These findings agree with previous studies that emphasize the hybrid behavior of titanate-based adsorbents^27^.

### Kinetics modeling

**Fig. 11:**
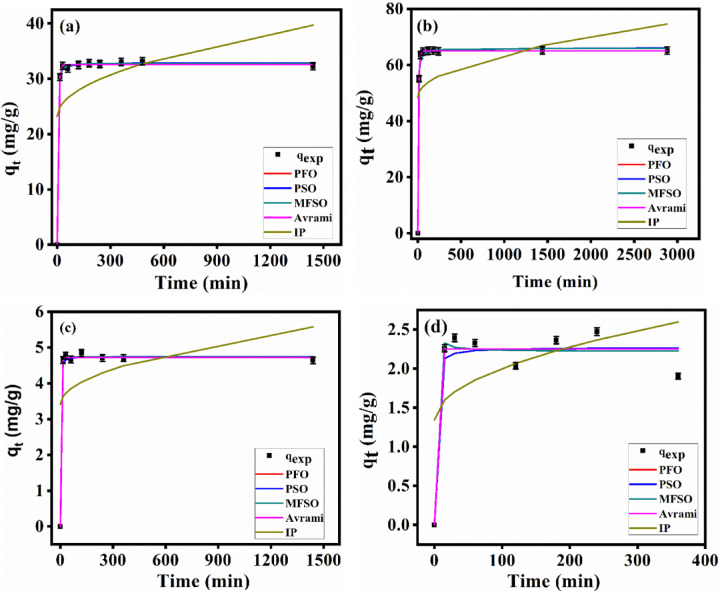
The adsorption kinetic modeling of the adsorption of Cs^+^ at initial concentrations 10 (**a**) and 20 (**b**) mg/L and Sr^+ 2^ at initial concentrations 10 (**c**) and 20 (**d**) mg/L onto TNTs using PFO, PSO, MFSO, Avrami and I-P models.

The kinetics of the radionuclide’s adsorption by TNTs and alginate-based materials are commonly investigated using various models. Figure 11 shows the kinetics of adsorption Cs^+^ and Sr^2+^ onto TNT using PFO, PSO, MFSO, Avrami, and I-P models. These kinetic models give valuable details about the potential rate-controlling step and mechanism of the adsorption process. Assuming the expected q_e_ and R^2^ values, the calculated q_e_ values from these models were close to the experimental results. So, it was suggested that the experimental results match the adsorption data well using the PFO, PSO, MFSO, and Avrami models. As a result, the process’s limiting rate includes physical and chemical interaction, which defines the dynamic behavior of both metal ions and included valence forces via electron exchange or sharing of electrons between adsorbent and metal ions. On the other hand, the I-P diffusion model doesn’t fit the adsorption of Sr^2+^ and Cs^+^ onto TNT because the R^2^ are very low (Table 2). Based on the error analysis (Table S2), MFSO model was the best to describe the adsorption of Cs^+^ at low concentration (10 mg/L) and Avrami was the best to describe Cs@T adsorption system at high concentration (20 mg/L). For Sr^2+^, MFSO was the best to describe the adsorption of Sr^2+^ at low concentration while PFO and Avrami were more accurate at high concentration (20 mg/L). On the other hand, I-P model failed to describe the adsorption systems under study at both concentrations.

**Table 2:** The values of the parameters of the adsorption kinetic modeling of Cs^+^ and Sr^2+^ onto TNTs using PFO, PSO, MFSO, avrami and I-P models along with the corresponding to R^2^ values.

Model	Parameters	Cs^+^		Sr^2+^	
		10 mg/L	20 mg/L	10 mg/L	20 mg/L
PFO	q_e_	2.25	4.72	32.61	65.02
	k_1_	0.698	0.246	0.177	0.125
	R^2^	0.95	0.99	0.99	1
PSO	q_e_	2.27	4.75	32.89	66.05
	k_2_	0.446	0.329	0.027	0.006
	R^2^	0.94	0.99	0.99	0.99
MFSO	q_e_	2.22	4.74	32.77	65.93
	k_t_	0.0003	0.239	0.014	0.002
	f_2_	1.0002	0	0.982	0.994
	R^2^	0.95	0.99	0.99	0.99
Avrami	q_e_	2.25	4.72	32.61	65.02
	k_av_	3.398	0.598	0.433	0.364
	n_av_	1.619	0.430	0.409	0.344
	R^2^	0.95	0.99	0.99	0.99
I-P	k_ip_	0.066	0.057	0.435	0.487
	c_ip_	1.34	3.41	23.19	48.47
	R^2^	0.27	0.17	0.22	0.16

### Proposed adsorption mechanism

1. Electrostatic Interactions

At the working pH range, the surface of TNTs carries negatively polarized –OH groups that attract positively charged Cs⁺ and Sr²⁺ ions. This electrostatic attraction constitutes the initial driving force for metal uptake and explains the rapid adsorption observed during the early contact period^28^. The slight changes in the FTIR –OH band intensity after adsorption confirm the participation of these groups in ion binding^29^.

2. Surface Complexation Mechanism

Following the initial attraction, surface coordination takes place between the radionuclides and oxygen-containing groups on the TNTs structure. The observed shifts in asymmetric and symmetric COO⁻ bands indicate that these groups participate in complexation with the hydrated metal ions^30^. Such interactions enhance adsorption stability and contribute to the PSO kinetic behaviour, which is characteristic of chemically influenced processes^31,32^.

3 Structural Rearrangement and Surface Modification.

After adsorption, FESEM images reveal slight surface roughening, suggesting localized modification of the TNTs exterior due to ion attachment. Additionally, BET analysis shows a small increase in surface area after adsorption, which can be attributed to ion-induced rearrangement of surface hydroxyl networks or opening of micro-channels within the titanate layers^33^. These structural changes further validate the mechanistic interpretation^34^.

4. Ion-exchange reactions.

The suggested mechanism of metals removal by TNTs is a mix between the ion exchange of Sr^+ 2^ and Cs^+^ with Na^+^ ion into Na_2_Ti_3_O_7_ as demonstrated by the pH results. The ion exchange of Sr^+ 2^ and Cs^+^ present in aqueous phase with the Na^+^ ions of Na_2_Ti_3_O_7_ can be expressed using Eqs. 4 and 5.

4 $${\mathrm{Na}}_{{\mathrm{2}}} {\mathrm{Ti}}_{{\mathrm{3}}} {\mathrm{O}}_{{\mathrm{7}}} \, + \,{\mathrm{2Cs}}^{ + } \rightleftharpoons {\text{ Cs}}_{{\mathrm{2}}} {\mathrm{Ti}}_{{\mathrm{3}}} {\mathrm{O}}_{{\mathrm{7}}} \, + \,{\mathrm{2Na}}^{ + }$$

5 $${\mathrm{Na}}_{{\mathrm{2}}} {\mathrm{Ti}}_{{\mathrm{3}}} {\mathrm{O}}_{{\mathrm{7}}} \, + \,{\mathrm{Sr}}^{{{\mathrm{2}} + }} \rightleftharpoons {\text{ SrTi}}_{{\mathrm{3}}} {\mathrm{O}}_{{\mathrm{7}}} \, + \,{\mathrm{2Na}}^{ + }$$

Therefore, considering that ion-exchange may be approximately as surface adsorption, it is expected that TNTs contain adsorption sites (ion-exchange sites) for Cs^+^ and Sr^[+ 2 4^, considering that the distribution coefficients are (K_d_). TNT had the highest K_d_ values for Cs^+^ and Sr^2+^ adsorption which will probably occur inside of nanotubes. The kinetics of ion exchange is an essential factor in determining the performance of the sorbent.

5. Integrated Mechanistic Pathway.

Overall, the removal of Cs⁺ and Sr²⁺ proceeds through three interconnected steps; (a) electrostatic attraction to surface –OH groups, (b) complexation and coordination with oxygen functionalities and (c) ion-exchange with structural Na⁺ in the titanate layers. This combined mechanism explains the strong affinity, high adsorption capacity, and rapid uptake rate observed for TNTs. The consistency between FTIR shifts, kinetic behavior, and isothermal modelling provides strong evidence supporting this multi-mechanistic interpretation.

### Regeneration performance and reusability assessment

The regeneration tests indicated that both TNTs and T/G composites were able to maintain a large fraction of their adsorption performance over several cycles. TNTs demonstrated remarkable stability, with less than a 10% decline in Cs⁺ and Sr²⁺ removal efficiency after five consecutive runs, which can be attributed to the robust ion-exchange properties of the layered titanate structure that preserves its framework under acidic conditions (Table 3). For the T/G composite, a slightly higher reduction in adsorption capacity, around 15%, was noted after five cycles, likely due to minor detachment of TNT particles from the alginate matrix during repeated rinsing. Despite this, the T/G beads remained structurally intact and easy to handle, confirming their suitability for practical batch or column applications. These observations align with earlier reports on the good reusability of titanate-based adsorbents and alginate-supported materials^7^.

**Table 3:** Summary of regeneration efficiency.

Cycle Number	TNTs		T/G		
	Cs⁺ Removal	Sr²⁺ Removal	Cs⁺ Removal	Sr²⁺ Removal	Notes
1	~ 100%	~ 100%	~ 100%	~ 100%	Baseline
2	97–98%	96–97%	95–96%	94–95%	Slight capacity loss
3	95%	94%	92%	90–91%	Stable performance
4	92–93%	92%	88–90%	87–88%	Minor degradation
5	90%	89–90%	85%	83–85%	Within acceptable limits

### Study limitations

From Table 4, the efficiency of the removal (R %) of Cs^+^ and Sr^2+^ by TNTs was in range of 85–96% while T/G is in range 60–90% which it is less because the ratio of TNTs to G is very low. Although, the introduction of alginate as a support to the TNTs reduces its efficiency in the removal of both metals. However, the composite still has the merit of applicability where it can be easily separated from water. Further study is required to improve the efficiency of the composites via optimizing the ratio of the TNTs to the alginate.

**Table 4:** Comparison of the removal efficiencies of radioactive metal from water using TNTs and T/G with reported literature.

Adsorbent mass	Solution pH	Adsorption time (min)	C_i_ (mg/L)	*R*% of Cs^+^		*R*% of Sr^2+^		Reference
				T	T/G	T	T/G	
0.075 g	8	30	10 mg/L	90	45	97	70	Current study
		60		88	32	96	85	Current study
0.15 g	6.5	120		85	60	99	90	Current study
20 mg	7–9	60	0.1 M	60				^3^

### Potential scalability and challenges

The ability to efficiently desorb ions using mild acidic solutions, along with the inherent structural stability of TNTs and the mechanical robustness of T/G beads, suggests that these materials could provide cost-effective and sustainable options for radionuclide removal. This feature significantly improves the practical applicability of the materials, addressing a common challenge associated with the repeated use of nanomaterial-based adsorbents in real-world applications. Incorporating TNTs into the alginate matrix reduced adsorption performance to 45–70% removal for Cs⁺ and 70–90% for Sr²⁺ under comparable conditions. Nevertheless, the T/G composite offered notable operational advantages, including mechanical stability, ease of separation, and suitability for handling scaled-up systems. From a scalability perspective, the fast adsorption kinetics, mild operating pH, and straightforward regeneration protocol support the feasibility of implementing TNT-based systems in practical remediation processes. Future studies should address optimization of TNT loading within the polymer matrix, evaluation under real wastewater conditions with competing ions, and long-term hydraulic stability in continuous-flow systems.

## Conclusion

This study provides a comprehensive assessment of titanate nanotubes (TNTs) and their alginate-based composite (T/G) for the removal of Cs⁺ and Sr²⁺ from aqueous media. The synthesized TNTs demonstrated rapid adsorption behaviour, achieving equilibrium within 15–30 min, with removal efficiencies reaching 90% for Cs⁺ and 97% for Sr²⁺ at pH 8, 0.075 g adsorbent dose, and an initial concentration of 10 mg/L. The structural and surface analyses revealed that metal uptake proceeded through a multi-pathway mechanism comprising electrostatic attraction, surface complexation, and partial ion exchange with Na⁺ ions in the Na₂Ti₃O₇ framework. Nonlinear isotherm modelling supported the heterogeneous nature of Cs⁺ and Sr^2+^ adsorption, with Sips and Langmuir–Freundlich (L–F) model providing the best fit and showing minimal error values (SSE, χ², and RMSE). TNTs exhibited maximum adsorption capacities (q_max_) of ~ 62 mg/g for Cs⁺ and ~ 165 mg/g for Sr²⁺, indicating excellent sorption capability. Regeneration studies confirmed the reusability of both adsorbents over multiple cycles. TNTs retained more than 90% of their initial capacity after five regeneration cycles, with performance loss remaining below 10%, while T/G displayed a moderate reduction of ≤ 15%, attributed primarily to minor TNT detachment from the alginate matrix. These outcomes confirm the structural robustness of TNT-based materials and their potential for water treatment.

## Supplementary Information

Below is the link to the electronic supplementary material.

Supplementary Material 1

## Data Availability

All data listed or discussed during this work are included in this published article and its supplementary information files.
