# Supplementary material for: Efficient removal of Cs+ and Sr2+ from water using titanate nanotubes embedded in alginate macromolecules
Source: Sci Rep. 2026 Feb 20;16:7483. doi: 10.1038/s41598-026-38030-8 (PMC12929695; doi:10.1038/s41598-026-38030-8)
Supplement: Supplementary file 1 — Supplementary Material 1 [file 41598_2026_38030_MOESM1_ESM.docx]

**Efficient removal of Cs^+^ and Sr^2+^ from water using Titanate nanotubes embedded in alginate macromolecules**

1. Adsorption isotherm modelling

1.1 Classical Langmuir (L)

This model (Eq. S1) predicts that the adsorption of the solid happens onto a homogenous surface in monolayer form with no interference between adsorbed ions because of a finite number of equivalent sites ^1^.

$q_{e}=\frac{q_{\text{m }K_{L}C_{e}}}{\left( 1+K_{L}C_{e} \right)}$ (nonlinear) (S1)

Where q_m_ is the maximum adsorption capacity of Cs^+^ or Sr^2+^ in mg/g. K_L_ is the affinity coefficient of the absorbent toward the adsorbate which is equivalent to the intensity of adsorption. This model may be related to an alteration of the adsorbent's suitable area and porosity, suggesting that a larger pore volume and surface area would lead to in a higher adsorption tendency ^2^. The separation factor R_L_ (Eq. S2) is a dimensionless constant that expresses the essential descriptions of the L isotherm.

R_L_=1/(1+K_L_C_o_) (S2)

Which 𝑅_𝐿_ values show the adsorption to be irreversible at R_L_= 0, linear at 𝑅_𝐿_ = 1, favorable at 0 < R_L_ <1, and unfavorable at R_L_ > 1 ^2^.

- 1. Freundlich isotherm (F)

Freundlich model (Eq. S3) provides an empirical representation of adsorption on surfaces that exhibit non-uniform energies. It accounts for the possibility of multilayer adsorption and reflects how adsorption intensity changes based on surface heterogeneity ^2^.

$q_{e}=K_{F}$ ${C_{e}}^{\frac{1}{\mathrm{nf}}}$ (nonlinear) (S3)

Where K_F_ is a coefficient related to the multilayer adsorption and 1/n_f_ is adsorption intensity. However, if 1/n_f_ = 0 shows that the adsorption is irreversible; 0 < 1/n_f_ < 1 this indicates a favorable adsorption process and 1/n_f_ > 1 indicates that adsorption is unfavorable ^3^.

1.3 Langmuir-Freundlich isotherm (L-F)

Langmuir–Freundlich model (Eq. S4) combines features of both Langmuir and Freundlich isotherms, offering a flexible description suitable for heterogeneous surfaces. It allows the adsorption system to transition between monolayer and multilayer behavior depending on the concentration. The constants of the model illustrate the maximum attainable capacity and the affinity of the adsorbent surface toward the ions ^2^.

q_e_ = (q_LF_ (K_LF_ C_e_) ^M^) / (1+ (k_LF_ C_e_) ^M^) (S4)

Where 𝐾_LF_ is the equilibrium constant for heterogeneous solids, and 𝑀is parameter with a value between 0 and 1. 𝑞_LF_ is the maximum adsorption capacity of L-F model (mg /g). The Langmuir constant k_LF_ indicates the adsorbate's affinity for the adsorbent surface.

1.4 Temkin isotherm

The Temkin isotherm incorporates the influence of indirect interactions between adsorbed species and the surface (Eq. S5). It assumes that the heat of adsorption decreases gradually as adsorption proceeds, reflecting a reduction in available high-energy binding sites. The parameters derived from this model provide insight into the strength of adsorbate–adsorbent interactions and the energy distribution over the surface ^4^.

q_e_= B_T_ ln(A_𝑇_).C_e_ (S5)

Where A_T_ (L/mg) and B_T_ (J/mol) are the constants of Temkin isotherm owned to the maximum binding energy and the sorption heat, respectively ^5^.

1.5 Dubinin-Radushkevich (D-R)

The D–R isotherm (Eq. S6) describes adsorption on energetically diverse surfaces and is often applied to evaluate adsorption in microporous materials. Rather than assuming a uniform surface, this model considers variations in adsorption energies, which can reflect multilayer formation ^2^. It is also useful for distinguishing between physical and chemical adsorption by estimating the mean adsorption energy. A lower value typically indicates physisorption, whereas higher values suggest stronger chemical interactions within the adsorbent structure ^4^.

q_e_ = q_m_ e ^− βε2^ (nonlinear) (S6)

Where β (mol^2^ kJ^−2^) is D-R isotherm constant and it is related to the adsorption energy, ε is the Polanyi potential depend on the concentration at equilibrium. E (kJ /mol) is the mean free energy of sorption (*E*) transferred from the solution onto the surface of the adsorbent ^5^. Equation S7 can be used to calculate *E*.

E = (1­/ ((2β) ^0.05^) (S7)

The process will be chemisorption if the value of the *E* equals 8 < E < 16 kJ /mol; physisorption occurs if it is less than 8 kJ /mol ^4^.

1.6 Sips

Sips isotherm (Eq. S8) is a mix of Langmuir and Freundlich isotherms models, and it has the following form:

q_e_ = (q_m_ K_s_C_e_^1/ns^) / (1+ K_s_C_e_^1/ns^) (S8)

Where K_s_ Sips constant [Lg^−1^], 1/n_s_ heterogeneity factor ^6^. The difference between the Sips and Langmuir isotherm equation is the heterogeneity factor 1/n_s_, generally ˂ 1. If this parameter’s value becomes smaller, the more heterogeneous the adsorbent surface will be. While 1/n_s_ is equal to 1, Sips isotherm equation is becomes Langmuir isotherm equation and the surface is homogeneous ^7^.

1.7 Redlich-Peterson (R-P)

The R-P isotherm model equation (Eq. S9) is based on the L-F equation. It is empirical and includes three parameters. Its mechanism of adsorption is a combination of both Langmuir and Freundlich model equations ^8^.

q_e_ = (K_R_ C_e_) / (1+a_R_ C_e_^β^) (S9)

Where K_R_ (L/g) and a_R_ (L/mg) are the constant of R-P isotherm, and β is an exponent in R-P isotherm and it is ranging from 0 and 1 ^9^. At high adsorbate liquid-phase concentrations, indicated to Freundlich isotherm model equation:

q_e_ = (K_R_/a_R_) * C_e_ ^1-β^ (S10)

In Freundlich isotherm model, K_R_/a_R_ = 𝐾_𝐹_ and (1−𝛽) = 1/𝑛. When 𝛽 = 1, reduces to Langmuir isotherm model equation with 𝑏 = 𝐵 Langmuir adsorption constant (L/mg) that corresponds to the adsorption energy.

1.8 Toth

Toth isotherm (Eq. S11) is also another modified form of Langmuir equation that aims to lower the error between experimental data and expected value of equilibrium data. It is especially suitable for characterizing heterogeneous adsorption systems that perform both the low and high-end adsorbate concentration boundaries ^10^.

q_e_ = (q_max_C_e_)/(1+(K_l_C_e_)^n^)^1/n^ (S11)

Where K_l_ is Toth constant in (mg/g) ^6^. The parameter 𝑛 characterizes the heterogeneity of the adsorption system. When n = 1, this equation reduces to Langmuir isotherm equation and if it deviates further away from unity (1), then the system is said to be heterogeneous.

1.9 Kahn

Kahn model (Eq. S12) is common for biadsorbate adsorption use of nonlinear methods from pure dilute equations solutions:

q_e_ = q_m_ b_k_C_e_/ (1 + b_k_C_e_) ^𝑎𝑘^ (S12)

Where 𝑎_𝑘_ and 𝑏_𝑘_ are Khan isotherm model exponent and constant, respectively ^10^.

1.10 Baudu

Baudu demonstrated that determining the Langmuir coefficients, k_L_ and q_m_, by measuring tangents at various equilibrium concentrations indicates that they are not constants in a wide range. As a result, Langmuir isotherm has been reduced to Baudu isotherm where b_o_ is the constant equilibrium, x, y are Baudu parameters. At lower surface coverage Baudu model reduces to Freundlich isotherm model. Nonlinear regression (Eq. S13) analysis is used to determine this isotherm's parameters because of the inherent bias caused by linearization ^11^.

q_𝑒_ = (𝑞_m_𝑏𝑜𝐶_e_^1+𝑥+𝑦^) / (1 + 𝑏𝑜𝐶_𝑒_^1+𝑥^) (S13)

1.11 Fritz-Schlünder (F-S)

Fritz-Schlünder is a five-parameter empirical model (Eq. S14) that may simulate the model modifications more accurately for use over a wide range of equilibrium data.

q_e_ = (q_mfs_ K_1_C_e_^m1^)/(1+K_2_C_e_^m2^) (S14)

Where m1, m2, K_1_, and K_2_, are F-S parameters and q_mfs_ is the maximum adsorption capacity in mg/g. F-S model reduces to Langmuir isotherm model if m1 and m2 = 1, yet it reduces to Freundlich isotherm model at large adsorbate concentrations. Nonlinear regression analysis may be used to find F-S isotherm parameters ^11^.

1. Kinetics study

Kinetic investigations are performed in batches, with non-linear and/ regression equations to establish the most suitable kinetic model.

2.1 Pseudo 1^st^ order model (PFO)

Pseudo 1^st^ model (Eq. S15) is utilized to determine the adsorption rate depending on the adsorption capacity ^12^, as it shown in Eq. S15

q_t_ = q_e_ (1-$\boldsymbol{e}^{\boldsymbol{-}\boldsymbol{t}\boldsymbol{*k}}$^1^) (S15)

Where q_t_ is the capacity of adsorption at time t (mg /g), q_e_ amount adsorbed at equilibrium (mg/ g), k_1_ (min^−1^) is the PFO rate constant.

2.2 Pseudo 2^nd^ order model (PSO)

The PSO model expects adsorption performance over the whole adsorption range and confirms that the main mechanism is the chemisorption ^12^, as it shown in Eq. S16

q_t_ = $\frac{k_{2}q_{e}^{2}t}{1+k_{2}q_{e}t}$ (S16)

Where k_2_ (g/ mg.min) is PSO rate constant.

2.3 Mixed 1^st^, 2^nd^ order model (MFSO)

The model ^13^ may be described as Eq. S17.

q_t_ = q_e_ $\frac{1-\exp(-k_{t})}{1-f_{2}exp (-k_{t})}$ (S17)

Where k_t_ is the adsorption rate constant (mg /g. min), and f_2_ is MFSO coefficient.

2.4 Avrami model

Avrami model ^14^ can be described by Eq. S18.

q_t_ = q_e_ (1-exp(-K_av_t$\boldsymbol{)}^{\boldsymbol{n}}$^av^  (S18)

Where K_av_ (min ^-1^) and n_av_ (-) are the constant and component of Avrami model.

2.5 Intraparticle diffusion model (I-P)

Intraparticle diffusion model (Eq. S19) illustrates the movement of species in solution from the bulk to the solid phase of porous material ^15^. It is the best way to describe adsorption that is controlled by adsorbate ion diffusion in the pores of porous adsorbents ^16^.

q_t_ = K_id_ t^1/2^+ C_x_ (S19)

Where k_id_ is the constant of I-P diffusion rate (mg/ g. min) and C_x_ refers to the boundary layer thickness ^15^.

3. Error Function Equations

The error functions used in this study were calculated using the following equations:

3.1 Sum of Squared Errors (SSE):

SSE = $\sum_{i=1}^{n} {(q}_{exp}-q_{cal})^{2}$ (S20)

A lower SSE value indicates a better model fit where q_exp_ (mg g⁻¹) and q_cal_ (mg g⁻¹) represent the experimental and model-calculated adsorption capacities at equilibrium, respectively; n is the total number of experimental data points; and i is the data index.

3.2 Chi-square (χ²):

χ² = $\sum_{i=1}^{n} \left( \frac{(q_{exp}-q_{cal})}{q_{cal}} \right)^{2}$ (S21)

A value close to zero indicates strong agreement between experimental and predicted values.

(3) Average Relative Error (ARE):

$\mathrm{ARE}=\frac{100}{n}+\sum_{i=1}^{n} \left( \frac{(q_{exp}-q_{cal})}{q_{cal}} \right)$ (S22)

ARE evaluates relative deviation; a lower value indicates better accuracy.

3.3 Root Mean Square Error (RMSE):

χ² = $\sum_{i=1}^{n} \left( \frac{(q_{exp}-q_{cal})}{q_{cal}} \right)^{2}$ (S23)

RMSE highlights the average magnitude of error.

Table S1. Summary for error analysis parameters for the adsorption isotherm modelling of Cs and Sr onto T/G nanocomposite

| Ion | Cs | | | | Sr | | | | |  |
| --- | --- | --- | --- | --- | --- | --- | --- | --- | --- | --- |
| Model | SSE | χ² | ARE (%) | RMSE | | SSE | χ² | ARE (%) | RMSE | |
| Langmuir | 205.6 | 22.30 | 38.10 | 4.53 | | 1504.02 | 217.66 | 46.99 | 9.70 | |
| Freundlich | 230.8 | 13.19 | 41.07 | 4.80 | | 3424.83 | 89.84 | 194.72 | 14.63 | |
| Dubinin-Radushkevich | 382.3 | 3.24E+13 | 59.49 | 6.18 | | 2418.22 | 4.42E+07 | 71.50 | 12.29 | |
| Temkin | 837.2 | 7.25 | 150.32 | 9.15 | | 2137.48 | -0.1009 | 185.59 | 11.56 | |
| Redlich-Peterson | 196.2 | 16.15 | 36.89 | 4.43 | | 800.72 | 46.13 | 67.27 | 7.07 | |
| Sips | 189.7 | 11.97 | 29.81 | 4.36 | | 645.75 | 37.75 | 82.37 | 6.35 | |
| Langmuir-Freundlich | 189.7 | 11.96 | 29.78 | 4.36 | | 645.75 | 37.75 | 82.37 | 6.35 | |
| Toth | 196.2 | 16.15 | 36.89 | 4.43 | | 800.72 | 46.12 | 67.27 | 7.07 | |
| Kahn | 198.2 | 17.81 | 37.85 | 4.45 | | 849.23 | 48.29 | 65.72 | 7.29 | |
| Baudu | 204.8 | 27.14 | 42.20 | 4.53 | | 879.94 | 49.27 | 66.94632 | 7.42 | |
| Fritz-Schlünder | 230.8 | 13.19 | 41.08 | 4.80 | | 3424.83 | 89.84 | 194.7237 | 14.63 | |

Table S2. Summary for error analysis parameters for the kinetics of Cs and Sr adsorption onto T/G nanocomposite

| Initial concentration 10 mg/L | | | | | | | | |  |
| --- | --- | --- | --- | --- | --- | --- | --- | --- | --- |
|  | **Cs** | | | | **Sr** | | | | |
| Model | **SSE** | **χ²** | **ARE (%)** | **RMSE** | **SSE** | **χ²** | **ARE (%)** | **RMSE** |  |
| PFO | 0.254 | 0.113 | 6.507 | 0.178 | 1.310 | 0.040 | 0.805 | 0.362 |  |
| PSO | 0.297 | 0.132 | 7.589 | 0.193 | 1.309 | 0.041 | 0.900 | 0.362 |  |
| MFSO | **0.246** | **0.110** | **6.700** | **0.175** | **1.254** | **0.039** | 0.900 | **0.354** |  |
| Avrami | 0.254 | 0.113 | 6.511 | 0.178 | 1.310 | 0.040 | 0.805 | 0.362 |  |
| I-P | 3.449 | 2.214 | 15.774 | 0.657 | 742.234 | 30.203 | 11.868 | 8.615 |  |
| Initial concentration 20 mg/L | | | | | | | | |  |
|  | **Cs Sr** | | | | | | | |  |
| Model | **SSE** | **χ²** | **ARE (%)** | **RMSE** | **SSE** | **χ²** | **ARE (%)** | **RMSE** |  |
| PFO | 0.030 | 0.006 | 1.042 | 0.062 | **0.131** | **0.002** | **0.142** | **0.121** |  |
| PSO | 0.053 | 0.011 | 1.446 | 0.082 | 12.491 | 0.205 | 1.447 | 1.178 |  |
| MFSO | 0.031 | 0.007 | 1.118 | 0.063 | 12.125 | 0.199 | 1.423 | 1.161 |  |
| Avrami | **0.029** | **0.006** | **0.981** | **0.060** | **0.131** | **0.002** | **0.1422** | **0.121** |  |
| I-P | 16.194 | 4.541 | 14.000 | 1.423 | 3081.993 | 61.766 | 12.344 | 18.505 |  |

**References**

[1] Goto, T., Cho, S. H., Lee, S. W. & Sekino, T. Sorption capacity of Cs+ on titania nanotubes synthesized by solution processing. *J. Ceram. Soc. Japan* **126**, 801–807 (2018).

[2] Ayawei, N., Ebelegi, A. N. & Wankasi, D. Modelling and interpretation of adsorption isotherms. *J. Chem.* **2017**, (2017).

[3] Tan, I. A. W., Ahmad, A. L. & Hameed, B. H. Adsorption of basic dye using activated carbon prepared from oil palm shell: batch and fixed bed studies. *Desalination* **225**, 13–28 (2008).

[4] Alharby, N. F., Almutairi, R. S. & Mohamed, N. A. Adsorption behavior of methylene blue dye by novel crosslinked O-CM-Chitosan hydrogel in aqueous solution: Kinetics, isotherm and thermodynamics. *Polymers (Basel).* **13**, 3659 (2021).

[5] Prihatdini, R. W., Suratman, A. & Siswanta, D. Linear and nonlinear modeling of kinetics and isotherm of malachite green dye adsorption to trimellitic-modified pineapple peel. *Mater. Today Proc.* **88**, 33–40 (2023).

[6] Serafin, J., Sreńscek-Nazzal, J., Kamińska, A., Paszkiewicz, O. & Michalkiewicz, B. Management of surgical mask waste to activated carbons for CO2 capture. *J. CO2 Util.* **59**, 101970 (2022).

[7] Sun, S. *et al.* 0D/1D Bi2O3@ TNTs composites synthesized by the decoration of Bi2O3 quantum dots onto titanate nanotubes: Synergistic adsorption of U (VI) and tetracycline. *Sep. Purif. Technol.* **306**, 122537 (2023).

[8] Brouers, F. & Al-Musawi, T. J. On the optimal use of isotherm models for the characterization of biosorption of lead onto algae. *J. Mol. Liq.* **212**, 46–51 (2015).

[9] Kumar, A., Kumar, S., Kumar, S. & Gupta, D. V. Adsorption of phenol and 4-nitrophenol on granular activated carbon in basal salt medium: equilibrium and kinetics. *J. Hazard. Mater.* **147**, 155–166 (2007).

[10] Padder, M. S. & Majunder, C. B. C. Studies on Removal of As (II) and S (V) onto GAC. *MnFe, 804 Compos. Isotherm Stud. Error Anal.* (2012).

[11] McKay, G., Mesdaghinia, A., Nasseri, S., Hadi, M. & Aminabad, M. S. Optimum isotherms of dyes sorption by activated carbon: Fractional theoretical capacity & error analysis. *Chem. Eng. J.* **251**, 236–247 (2014).

[12] Revellame, E. D., Fortela, D. L., Sharp, W., Hernandez, R. & Zappi, M. E. Adsorption kinetic modeling using pseudo-first order and pseudo-second order rate laws: A review. *Clean. Eng. Technol.* **1**, 100032 (2020).

[13] Shehata, N., Faisle, M., Farghali, A., EL-Rabiei, M. & Mahmoud, N. Kinetic studies for the remediation of some pollutants from real wastewater onto carbon nanotube. *J. Environ. Eng. Sci.* **17**, 108–119 (2022).

[14] Avrami, M. Kinetics of phase change. I General theory. *J. Chem. Phys.* **7**, 1103–1112 (1939).

[15] Dakroury, G. A., El-Shazly, E. A. A. & Hassan, H. S. Preparation and characterization of ZnO/Chitosan nanocomposite for Cs (I) and Sr (II) sorption from aqueous solutions. *J. Radioanal. Nucl. Chem.* **330**, 159–174 (2021).

[16] Sutirman, Z. A., Sanagi, M. M. & Aini, W. I. W. Alginate-based adsorbents for removal of metal ions and radionuclides from aqueous solutions: A review. *Int. J. Biol. Macromol.* **174**, 216–228 (2021).
